# “Remodeling the intestinal immune microenvironment”: immune regulation and tissue regeneration by mesenchymal stem/stromal cells in the repair microenvironment of inflammatory bowel disease

**DOI:** 10.3389/fimmu.2025.1543702

**Published:** 2025-05-13

**Authors:** Hongkun Li, Yuyue Zhang, Simin Du, Jinghan Shen, Xingyan Liu, Jie Jing

**Affiliations:** School and Hospital of Stomatology, Zunyi Medical University, Zunyi, Guizhou, China

**Keywords:** inflammatory bowel disease, mesenchymal stem/stromal cells, intestinal immune microenvironment, biological therapies, tissue regeneration

## Abstract

The global prevalence of inflammatory bowel disease (IBD) has significantly increased in recent decades. IBD is a long-term, recurring, gastrointestinal inflammatory condition that mainly comprises two primary clinical types: ulcerative colitis and Crohn’s disease. The current treatment paradigm for IBD primarily focuses on symptom management. However, this approach does not support mucosal epithelial repair, maintenance of barrier homeostasis, or regulation of biological functions in the gut. Conventional therapies rely on the frequent use of high-dose medications, including antibiotics, nonsteroidal anti-inflammatory drugs, biological agents, and immunomodulators. Recently, mesenchymal stem/stromal cells (MSCs) have gained interest in tissue regeneration owing to their unique ability to differentiate and secrete regulatory factors, including extracellular vesicles (EVs), which play crucial roles in abnormal organization. Various routes of administration have been explored in preclinical and clinical studies to deliver MSCs from diverse tissue sources. The routes include intraperitoneal, intravenous, and local (intracolonic or rectal) delivery. The MSCs employed were obtained from various tissues, including bone marrow, umbilical cord, and adipose tissue. This article reviews the research framework for the application of MSCs and EVs secretion in the treatment of IBD, emphasizing key immunological effects, such as immune microenvironment regulation, intestinal barrier stabilization, and therapeutic approaches targeting intestinal barrier disorders. The discussion primarily focuses on the advantages of MSCs over other biologics, impairment of gut mucosal tissue-resident mesenchymal stem cells in IBD development, immune targets (at the cellular and molecular levels) within the framework of IBD, and the reparative effects of MSCs in the microenvironment of IBD. We aimed to present an overview of the current trends in MSC research and therapy, as well as to identify the challenges and future directions that must be addressed to advance research on MSC-mediated therapeutic strategies for IBD.

## 1 Introduction

IBD comprises chronic, unexplained inflammatory conditions that affect the gastrointestinal tract. The two primary clinical types of IBD are ulcerative colitis (UC) and Crohn’s disease (CD). In the 21st century, IBD has emerged as a major global health issue, with its incidence rising in nearly every industrialized country, placing a significant burden on public health systems (1, 2). IBD typically manifests during adolescence and young adulthood, with its prevalence rising in the pediatric population. The pathogenesis of IBD is driven by genetic predisposition, dysregulated mucosal immune responses, and imbalances in the intestinal microbiota (3, 4).

Although the causes of IBD have been extensively investigated, they remain incompletely understood. Current insights into this complex condition suggest that its origin is multifactorial (5). It is hypothesized that endogenous triggers, including genetic susceptibility and immune system dysregulation, and external environmental triggers, such as microbial exposure, chemical exposure, psychological stress, and dietary factors, contribute to IBD development (6–9). Recent microbiome research has demonstrated that dysbiosis, characterized by alterations in the composition of the gut microbiota, contributes significantly to IBD development (10). Intestinal mucosal inflammation, epithelial damage, and the resulting imbalance in the intestinal mucosa further influence the etiology of IBD (11). These factors contribute to the complexity of IBD etiology.

Although the exact pathological mechanisms underlying IBD have not been fully elucidated, it is characterized by a complex interplay between genetic predispositions and environmental factors involving abnormal and sustained T cell-mediated immune responses targeting the commensal gut microbiota (12, 13). The combined effects of multiple factors lead to multilayered mucosal damage in the intestine, including histological and cellular changes, eventually resulting in fibrosis, macroscopic erosion, and ulceration (14).

The symptoms of IBD typically include weight loss, chronic diarrhea, abdominal pain, rectal bleeding, and strictures, leading to malnutrition and fatigue. UC and CD are also associated with an increased risk of colorectal cancer and a diminished quality of life due to the chronic and debilitating nature of the disease (15, 16). Furthermore, patients with IBD commonly present with extraintestinal manifestations (17). Growth retardation and oral mucosal ulcers are the most prevalent manifestations. IBD is frequently accompanied by a wide array of extraintestinal manifestations that affect multiple organ systems. Joint disorders such as arthritis and ankylosing spondylitis are among the most common, reflecting the systemic inflammatory nature of the disease. Dermatological manifestations, including erythema nodosum, characterized by painful skin nodules, and hepatobiliary conditions, such as primary sclerosing cholangitis (18), further demonstrate the multisystem involvement of IBD. Additionally, renal complications, including tubulointerstitial nephritis, arise from immune-mediated mechanisms or secondary effects of medication. Neurological complications, although less common, include peripheral neuropathy, headache, and cognitive dysfunction, which significantly affect patient outcomes (19). However, recurrent, progressive, and destructive inflammation caused by uncontrolled activation of intestinal immune cells in genetically susceptible hosts has the greatest impact on the quality of life of patients with IBD (20). These findings highlight the necessity of restoring normal growth, administering effective anti-inflammatory treatments, eliminating complications in children with IBD, and enhancing patients’ quality of life.

Improving patients’ quality of life is the primary aim of IBD treatment and is accomplished by achieving clinical, patient-reported, histological, or endoscopic remission (21). However, substantial variability in disease phenotypes and severity complicates the prediction of symptoms and treatment response (22). The current treatment strategies aim to achieve deep and durable remission to prevent complications, facilitate complete mucosal healing, normalize blood markers, and eliminate symptoms. This can be achieved through surgical intervention, which halts disease progression.

Implementation of early immunosuppressive therapy or its combination with biologics in high-risk patients is fundamental to these approaches. Additionally, maintaining strict and consistent inflammation control and adjusting treatments based on evaluations, known as treatment-targeting strategies, is essential. Consequently, the development of drugs capable of predicting treatment responses will become increasingly crucial for personalized medical decision-making in the near future.

Conventional IBD treatments, including 5-aminosalicylates (23) and corticosteroids, are recommended to induce and sustain remission in patients with mild-to-moderate disease. Systemic glucocorticoids (24) and immunosuppressants (25) are administered to alleviate symptoms in patients with moderate-to-severe UC. However, high-quality evidence supporting the effectiveness of these treatments for CD remains lacking. High-risk patients require treatment with biologics (26, 27), with or without concomitant immunomodulatory agents to maintain remission. However, surgical intervention is mandatory in some patients. Nevertheless, in most cases, surgical intervention is not the definitive solution, necessitating continued administration of medical therapy to prevent CD recurrence (28). Beyond the limited efficacy of standard treatments, it is crucial to recognize the potential for severe side effects, including drug-induced fever, skin rashes, and neurotoxicity (29). High recurrence rates and contraindications associated with IBD restrict the applicability of numerous conventional therapeutic modalities. Over the past two decades, the advancement and utilization of biologics (30) have spurred extensive research into the incorporation of biopharmaceutical agents alongside traditional medications for the treatment of IBD. Treatment approaches for IBD, particularly moderate-to-severe cases, have evolved substantially. Biopharmaceutical agents have demonstrated greater efficacy than conventional pharmaceuticals in managing IBD.

## 2 Advantages of biologic agents compared to traditional pharmacotherapy

In the current clinical management of IBD, biologics provide patients with personalized therapeutic approaches. Soluble protein ligands, particularly inflammatory cytokines, serve as crucial targets in drug development, driving the advancement of therapies such as anti-leukocyte trafficking drugs, tumor necrosis factor (TNF) inhibitors (31), integrin inhibitors, and adhesion molecule blockers (32). Their central role in immune modulation and involvement in chronic inflammatory diseases underscores their therapeutic significance. These biologics primarily exert their effects by negatively regulating the immune system and modulating the inflammatory pathways that drive disease. Currently, the most frequently used biologics for the clinical management of IBD include TNF, integrin, and interleukin (IL)-12/23 inhibitors (33).

Traditional medications such as aminosalicylates (e.g., sulfasalazine and mesalazine), corticosteroids (e.g., budesonide and prednisone), and immunosuppressants (e.g., azathioprine) exert their therapeutic effects by broadly suppressing the immune system, which may lead to systemic side effects (34). Conversely, biologics more precisely target the immune molecules implicated in IBD, including TNF-α, various ILs, and integrins. Although certain biologics, particularly TNF inhibitors, are associated with risks such as severe infections, lymphoma, and other malignancies, their targeted mechanism of action limits widespread immunosuppression, thus mitigating systemic adverse effects (35). Biologics can reduce or eliminate severe side effects, such as weight gain, osteoporosis, and diabetes, which are often induced by prolonged corticosteroid therapy. For patients experiencing significant adverse reactions or inadequate responses to conventional treatments (e.g., steroids, thiopurines, or methotrexate), biologics offer a safer alternative. Studies have demonstrated that biologics can delay or obviate the need for surgical intervention, thereby reducing the demand for surgical procedures in patients with IBD (36, 37). Consequently, the benefits of biologics frequently outweigh their risks when administered to appropriately selected patients. Research has validated their clinical efficacy, and well-established dosing regimens, dose adjustment protocols, and monitoring procedures have been developed (38).

Extensive research encompassing animal studies, clinical trials, and meta-analyses has substantiated significant advances in biological therapy, particularly in enhancing long-term disease management, inducing remission, and improving patient quality of life. Biologics such as TNF inhibitors (e.g., infliximab and adalimumab) and integrin inhibitors have demonstrated unequivocal efficacy in inducing and maintaining remission in moderate-to-severe IBD, reducing the frequency of flare-ups and hospitalizations (39, 40). Studies indicate that these agents rapidly and effectively alleviate symptoms such as abdominal pain, diarrhea, and fatigue while promoting mucosal healing and mitigating complications (e.g., strictures, fistulas, and intestinal perforation) (41–43). These clinical improvements enable patients to resume normal activities and enhance their overall quality of life.

### 2.1 TNF-α inhibitors

TNF−α inhibitors, including infliximab, adalimumab, and golimumab, have been utilized in clinical practice for many years. These agents specifically target TNF−α, a cytokine that plays a central role in the inflammatory processes underlying IBD. By inhibiting TNF−α, they block pro-inflammatory signaling pathways, induce T-cell apoptosis and stimulate the production of anti-inflammatory cytokines, thereby inducing and maintaining remission in both CD and UC (39). TNF−α inhibitors remain a valuable treatment option for patients with moderate-to-severe IBD who do not respond adequately to conventional therapies. Their use can decrease the need for corticosteroids and promote mucosal healing. In clinical practice, infliximab and adalimumab are among the most well-established and widely used biologics, with adalimumab often employed following a loss of response to infliximab (43, 44). Additionally, golimumab has demonstrated effectiveness in both CD and UC, frequently providing a more durable response (45). Nevertheless, several challenges remain. Primary nonresponse and the development of anti-drug antibodies (ADAs), owing to the intrinsic immunogenicity of these agents, can diminish treatment efficacy or trigger hypersensitivity reactions. These reactions may include infusion- or injection-related responses characterized by fever, rash, and chills. Moreover, TNF−α inhibitors have been linked to serious adverse events, such as malignancies and congestive heart failure (38, 46, 47).

### 2.2 Integrin inhibitors

Vedolizumab is a highly selective integrin inhibitor that targets the α4β7 integrin, preventing the homing of effector T lymphocytes and B cells to the gut mucosa. It is primarily used for managing UC and CD, particularly in patients unresponsive to TNF inhibitors (33). Its robust efficacy is largely attributable to its minimal systemic immunosuppression, and it outperforms anti-TNF-α therapy during the maintenance phase (48). Conversely, natalizumab is an earlier integrin inhibitor that targets the α4 integrin, resulting in a broader spectrum of activity compared to vedolizumab. It is predominantly indicated for CD, especially in refractory cases, because of its effectiveness in impeding the migration of inflammatory cells to the gastrointestinal tract (40, 49). However, the use of integrin inhibitors is associated with an increased risk of infections, including progressive multifocal leukoencephalopathy, with natalizumab specifically implicated in such adverse events (47). Ongoing clinical investigations, such as the GEMINI trial, are evaluating the long-term efficacy and safety of these agents (50). Moreover, the SEAVUE study is exploring the combination of vedolizumab with other therapies to enhance remission rates and improve overall patient outcomes (51).

### 2.3 IL-12/23 inhibitors

Ustekinumab, an IL-12/23 inhibitor, targets the p40 subunit shared by these cytokines, which is a critical component in the differentiation and activation of T cells into T helper 1 (Th1) and T helper 17 (Th17) subsets, as well as in recruiting monocytes and neutrophils (33). Ustekinumab effectively attenuates the inflammatory response in both UC and CD by antagonizing IL-12 and IL-23, thereby inducing clinical remission (52, 53). Furthermore, it has demonstrated robust efficacy in inducing and maintaining remission in patients who are refractory to other biologics or immunosuppressive therapies (54, 55). An ongoing UNIFI study is evaluating the long-term efficacy and safety of ustekinumab in UC treatment, with emerging data supporting its sustained benefits over time (56).

### 2.4 Janus Kinase inhibitors

Tofacitinib and Filgotinib are oral Janus Kinase (JAK) inhibitors that modulate the JAK-STAT signaling cascade, which is a pivotal pathway in the pathogenesis of UC and CD. Inhibiting this cascade leads to reduced IL-12 and IL-23 (57). Unlike previously discussed intravenous or subcutaneous biologics, these oral agents offer a less invasive therapeutic alternative, thereby mitigating infusion- or injection-related adverse events. Clinical trials have demonstrated their efficacy in both inducing and maintaining remission in patients with moderate-to-severe UC (58) and have demonstrated comparable effectiveness in patients with moderate-to-severe CD who are refractory or intolerant to TNF inhibitors (59). Both Tofacitinib and Filgotinib have been approved for the treatment of UC, particularly in individuals for whom other biological or conventional therapies have failed. However, their use is associated with an elevated risk of infection and thromboembolic events. Ongoing investigations continue to delineate their broader role in IBD and assess their long-term safety profiles (60).

### 2.5 Emerging targets and therapies

Novel biologics are currently being investigated in clinical trials to address the limitations of existing approved therapies. One such agent, the IL-23 inhibitor risankizumab, has demonstrated promising potential for the treatment of CD (61). IL-17 inhibitors, including brodalumab, have shown therapeutic effects against IBD. Despite the role of IL-17 in mucosal inflammation, reports suggest that its inhibitors may exacerbate IBD in certain cases (62). B cells are critical for the pathogenesis of IBD; however, the limitations of rituximab in depleting B cells have rendered it ineffective in clinical trials for IBD. Alternative strategies, such as plasma cell depletion, remain potential therapeutic avenues, although further robust studies are needed (63). Additionally, novel biologics, including new oral anti-TNF-α agents and innovative adhesion inhibitors, are under development (33).

## 3 Advantages of MSCs over other biologics

Contemporary clinical research increasingly supports the early initiation of biological therapies during the disease course. Furthermore, combining biologics with immunosuppressive or non-biological treatments may provide more comprehensive disease control. Receptor-targeted (64) or ligand-targeted (12) single drugs have relatively limited roles in treating IBD. Most of these drugs require the use of combination therapies or delivery systems, and current therapeutic modalities are inadequate for preventing and alleviating intestinal fibrosis and stenosis in IBD (65–67). The absence of effective anti-fibrotic drugs has made surgical intervention the primary strategy for managing intestinal fibrosis and stenosis in patients with IBD (68–70). Additionally, the long-term efficacy of these treatments is compromised by the accumulation of toxic side effects (71).

Most of these biological products expose patients to opportunistic infections and immunosuppression-related adverse effects. Clinical studies have demonstrated that many patients receiving biological therapies experience either primary nonresponse or secondary loss of response owing to inadequate drug concentrations or the development of ADAs. These phenomena can result in reduced therapeutic efficacy, infusion-related reactions, and hypersensitivity, potentially compromising the long-term effectiveness of biologics (38, 72, 73). Moreover, biological therapies are associated with an elevated risk of infections, malignancies, and cardiovascular events, and their prolonged use may further impair immune function, leading to additional complications. These treatment strategies have limited efficacy in addressing issues such as mucosal barrier homeostasis and intestinal biological dysregulation (11). Recent studies have increasingly focused on tailoring biological therapies to individual patients based on biomarkers and genetic characteristics. Advances in pharmacogenomics and molecular diagnostics have enabled clinicians to predict which patients will benefit most from specific biologics, facilitating personalized and effective treatment strategies (38).

Although biologics can elicit immune responses that lead to ADA formation, autologous MSCs are less likely to provoke such immunogenic reactions, reducing the risk of adverse effects and treatment resistance associated with biological therapies. MSC therapy shows promise for inducing long-term remission and, in some cases, achieving a cure, thereby reducing the need for recurrent biological treatments. Moreover, allogeneic (donor-derived) MSCs are readily available, broadening the range of therapeutic options. MSCs are generally considered safe and have demonstrated a favorable safety profile in clinical trials (74), with a lower incidence of severe side effects, such as infections or malignancies, compared to some biological treatments.

In recent years, MSCs have emerged as a critical area of research in tissue repair and regeneration owing to their unique ability to differentiate, home to specific sites (75), and secrete regulatory factors, particularlyEVs (76). Evidence suggests that bone marrow MSCs (BM-MSCs) (77), umbilical cord MSCs (UC-MSCs) (78), Adipose tissue-derived MSCs (AT-MSCs) (79) and tissue-resident MSCs (TR-MSCs) (80) play crucial roles in repairing IBD, thereby advancing strategies for tissue regeneration. Although the risks associated with MSC therapy are still under evaluation, current evidence indicates that these therapies are clinically safe. Consequently, MSCs have become a cornerstone of regenerative medicine.

## 4 Characterization of MSCs

MSCs are multipotent stem cells capable of differentiating into osteocytes, chondrocytes, adipocytes, and other cell types, offering significant potential for tissue regeneration (81). In addition to these mesenchymal cell lines, MSCs have been reported to generate other cell types, including epithelial cells (82). Furthermore, MSCs exhibit immunomodulatory capabilities and tumor-homing properties (83, 84), making them valuable tools for managing numerous pathological processes.

Exosomes, a subclass of EVs secreted by MSCs, are pivotal in mediating their therapeutic benefits. These nanoscale lipid-bound vesicles carry diverse bioactive molecules and are distinguished by their biogenesis via the endosomal pathway and unique molecular composition. MSCs influence tissue repair through paracrine factors (85), and MSC-derived exosomes (MSC-Exos) transport complex proteins, nucleic acids, and lipids and contain abundant molecules such as microRNAs (miRNAs), pro-inflammatory cytokines, and anti-inflammatory cytokines (**Table 1**). MSC-Exos prevents miRNA degradation in body fluids by ribonucleases (105) and delivers their content to recipient cells (106). Additionally, exosomes function as natural nanocarriers capable of transporting various biomolecules, including functional RNA, proteins, synthetic drugs, and therapeutic agents. They can be engineered to perform expanded functions using various methods. Recent studies indicate that MSCs can elevate the levels of immunosuppressive molecules, such as IL-10 and transforming growth factor-β (TGF-β), in the colon (107) and upregulate the expression of miR-125a and miR-125b (108), contributing to the amelioration of IBD. Owing to their unique properties, MSC-Exos have significant potential for clinical applications in tissue repair and regeneration (109), as well as in disease diagnosis and prognosis (110).

**Table 1:** Broad Spectrum of Biological Regulatory Molecules Secreted by MSCs and Their Regulatory Effects on Inflammation.

Source	Mediator	Processes and Effects	References	Pro-inflammatory or Anti-inflammatory
Cytokines				
Mouse BM-MSCs	IL-10	Lipopolysaccharide-activated MSCs promote the expression of NOD-like receptor protein 3 and the production of IL-10	(86)	Anti-inflammatory
AD-MSCs-derived EVs	TSG-6	TSG-6 in EVs is a major factor in the relief of dextran sodium sulfate-induced colitis, by increasing the number of Tregs and macrophage polarization from M1 to M2 in the colon	(87)	Anti-inflammatory
hMSC	PGE2、TSG-6	MSCs reduce the production of TNF-α, IL-1, and IL-6 in macrophages and increase the production of IL-10 by secreting soluble factors such as PGE2 or TSG-6	(88, 89)	Anti-inflammatory
TNFα-stimulated MSCs	TIMP2,TGF-β1,HGF、Kynurenine (produced via indoleamine2,3-dioxygenase activity)		(90)	Anti-inflammatory
BM-MSCs	IGF-1、TGF-β1		(91)	Anti-inflammatory
AD-MSCs	IL-6、IL-8、IP-10		(92)	Pro-inflammatory
Human thymic mesenchymal stromal cells	TSP-1	Exosomes derived from Lipopolysaccharide-pretreated thymic mesenchymal stromal cells promoted the polarization of macrophages to M1-like phenotype and the secretion of IL-6 and TNF-α, as well as the pro-inflammatory differentiation of CD4+T cells to Th17 cells through TSP-1	(93)	Pro-inflammatory
microRNA				
hUC-MSCs	miR-24-3p	MSC-Exos exhibit high enrichment of miR-24-3p, which modulates macrophage polarization through the targeted inhibition of the STING in macrophages	(94)	Anti-inflammatory
Mouse BM-MSC	miR-27 b	miR-27b is abundantly expressed in MSC-Exos and suppresses the expression of pro-inflammatory genes by preventing the recruitment of JMJD3 and NF-κB to the promoter region.	(95)	Anti-inflammatory
hUC-MSCs	miR-146a	IL-1β-pretreated MSCs selectively incorporate miR-146a into exosomes, which are subsequently transferred to macrophages, thereby promoting M2 polarization.	(96)	Anti-inflammatory
hBM-MSCs	miR-147b	The expression of miR-147B in MSC-Exos can inhibit the inflammatory effect by inhibiting the NF-κB pathway	(97)	Anti-inflammatory
Mouse BM-MSC	miR-182	MSC-Exos shuttling miR-182 promote the polarization of M1 macrophages towards M2 macrophages by targeting the TLR4/NF-κB/PI3K/Akt signaling cascade	(98)	Anti-inflammatory
hUC-MSC-derived EVs	miR-125 b 、 let-7b and miR-6873	hucMSC-derived EVs, through the enrichment of specific microRNAs, inhibit the activation of the IRAK1/TAB2/NF-κB signaling pathway and restore the normal expression levels of IL-4, IL-8, IL-10, IL-13, IL-17, and TNF-α	(99)	Anti-inflammatory
Pre-stimulation MSCs with TNF and IFN-γ	miR-7704	miR-7704 promotes M2 macrophage polarization by directly binding to MyD88 and subsequently inhibiting the MyD88/STAT1 signaling pathway	(100)	Anti-inflammatory
Human and mouse BM-MSCs	miR-21	MSCs within the glioma microenvironment upregulate miR-21 expression in MSC-Exos through a miR-21/SP1/DNMT1 positive feedback loop. miR-21 potentiates CD73 expression on MDSCs by activating the PTEN/PI3K/AKT/HIF-1α signaling pathway, thereby enhancing their immunosuppressive effects. Moreover, miR-21 promotes M2 macrophage polarization, stimulates the differentiation and activation of MDSCs, and suppresses T cell activation	(101)	Anti-inflammatory
Mouse BM-MSCs-derived EVs	miR-223	MSC-EVs attenuated liver injury via transfer of miR-223-3p which targeted proinflammatory gene STAT3 in macrophages	(102)	Anti-inflammatory
LPS-preconditioned hUC-MSCs	miR-let-7b	miR-let-7b regulates macrophage polarization through modulation of the TLR4/NF-κB/STAT3 signaling pathway	(103)	Anti-inflammatory
hUC-MSCs	miR148b-5p	hucMSCs exert anti-inflammatory effects through the release of miR-148b-5p, which inhibits the expression of 15-lipoxygenase-1 (15-LOX-1) in macrophages	(104)	Anti-inflammatory

EVs, extracellular vesicles; MSCs, Mesenchymal Stromal/Stem Cells; BM-MSCs, Bone marrow Mesenchymal Stromal/Stem Cells; AD-MSCs, Adipose tissue-derived Mesenchymal Stromal/Stem Cells; hMSC, Human Mesenchymal Stromal/Stem Cells; hUC-MSCs, Human Umbilical Cord Mesenchymal Stromal/Stem Cells; hBM-MSCs, Human Bone Marrow-Derived Mesenchymal Stromal/Stem Cells; M1, Classically activated macrophages; M2, Alternatively activated macrophages; Tregs, regulatory T cells; TNFα, tumor necrosis factor alpha; IFN-γ, interferon-gamma; IL, interleukin; TSG-6, tumor necrosis factor-inducible gene 6; PGE2, prostaglandin E2; TIMP, tissue inhibitors of metalloproteinases; TGF-β, transforming growth factor-beta; HGF, hepatocyte growth factor; IGF, insulin-like growth factor; IP-10, interferon γinducible protein-10; TSP-1, thrombin-sensitive protein,Thrombospodin-1.

Nevertheless, the cytokine and miRNA profiles of MSCs vary depending on their source and culture conditions. Research has demonstrated that exosomes derived from BM-MSCs contain 730 functional proteins, including those implicated in tissue regeneration, such as those involved in angiogenesis, coagulation, apoptosis, inflammation, and extracellular matrix remodeling (111). These findings suggested that MSCs play a pivotal role in modulating inflammatory responses, promoting intestinal repair, and mitigating fibrosis in patients with IBD.

MSC-Exos are enriched in various nucleic acids that regulate the function and activity of the target cells. Notably, the proportion of miRNAs in exosomes was significantly higher than that in parent cells, enabling them to modulate gene expression in recipient cells. Specific miRNAs such as miR-24-3p (94), miR-27b (95), miR-125b (112, 113), miR-146a (96), miR-147 (97), miR-182 (98), miR-6873 (99), and miR-7704 (100) exhibit anti-inflammatory properties. Additionally, miR-21 exerts both anti-inflammatory and anti-apoptotic effects, thereby contributing to tissue repair by promoting cell growth (101). Moreover, miR-223 modulates immune cell activation and reduces inflammation in various experimental models (102), and certain Let-7 family members downregulate pro-inflammatory gene expression (103, 114). Although miR-155 is recognized as a prominent pro-inflammatory mediator, its levels in MSC-Exos are generally low under standard conditions. MSCs may adopt a more pro-inflammatory miRNA profile when exposed to inflammatory signals, but this shift is not typically observed in MSC-Exos (115).

In addition to nucleic acids, MSC-Exos contains diverse lipid components that are crucial for the membrane structure and cell signaling. They are enriched in polyunsaturated fatty acids (PUFAs), particularly phosphatidylcholine, which is highly unsaturated. These PUFAs protect exosomes from free radical-induced damage and confer anti-inflammatory properties (116).

Key repair molecules within MSC-Exos include growth factors and cytokines such as TGF-β, hepatocyte growth factor (HGF), vascular endothelial growth factor, β-fibroblast growth factor (117), and insulin-like growth factor (IGF) (91), with TGF-β and HGF also demonstrating immunosuppressive effects. Anti-inflammatory cytokines include IL-10 (86), prostaglandin E2 (PGE2) (88), tumor necrosis factor-inducible gene 6 (TSG-6) (87, 89), IGF, kynurenine (produced via indoleamine2,3-dioxygenase activity), and tissue inhibitors of metalloproteinase 2 (90). Although MSC-Exos typically promote tissue repair through their anti-inflammatory actions, under certain conditions, they may also carry pro-inflammatory cytokines, including IL-6, IL-8, interferon-gamma (IFN-γ)-inducible protein-10 (IP-10) (92), and thrombospondin-1 (93).

Compared to MSCs, MSC-Exos are more readily stored and transported (118). Additionally, they exhibit low immunogenicity, thereby mitigating the potential risks associated with cell-based therapies (119). MSC-Exos play a pivotal role in mediating intercellular material transfer and facilitating signal transduction, thereby enhancing the communication between cells. When exosomes are released into the extracellular environment, they interact with target cells through the following mechanisms.

1. MSC-Exos enter target cells via endocytosis or direct fusion with the cell membrane (105).
2. MSC-Exos attach to the cell surface by interacting with lipid ligands on their receptors (120).
3. After binding to the target cells, MSC-Exos can directly or indirectly activate signal transduction pathways within these cells.

Therefore, MSC-Exos are more commonly utilized in preclinical and fundamental research than MSCs. Overall, MSCs have made only limited progress in translational medicine owing to the incomplete understanding of their controversial roles and the EVs they secrete in IBD. However, MSCs remain promising candidates for combating IBD-related complications and for further therapeutic advancements.

## 5 Therapeutic role of MSCs in IBD

In the presence of severe inflammation, MSCs can promote the formation of a balanced inflammatory and regenerative microenvironment in damaged tissues (121). Epithelial cells in the mucosal crypt area of patients with CD are susceptible to pro-inflammatory cytokines, and subepithelial muscle fibroblasts (MFs) of the intestinal mucosa in patients with IBD are destroyed and lost. The loss of MFs is attributed to their susceptibility to pro-inflammatory cytokines (122). MSCs may exert immunotherapeutic effects on IBD by enhancing MF function.

MSCs and MSC-Exos have demonstrated significant therapeutic potential in both preclinical studies and clinical trials using IBD models. These agents effectively regulate immune responses, attenuate inflammation, and enhance tissue repair, presenting a compelling and promising strategy for IBD treatment. MSCs exhibit a remarkable capacity to home to sites of injury, including the intestinal mucosa, where they perform the dual functions of modulating inflammatory responses and facilitating tissue regeneration (123). In many animal studies, MSCs were primarily administered via intraperitoneal and intravenous injections, followed by localized injections for disease treatment (124, 125). However, subsequent research has shown that MSCs do not need to colonize target tissues to exert their therapeutic effects (126). MSCs possess a homing ability, allowing them to migrate to injury sites (127, 128). Additionally, MSCs can differentiate into local components at the injured site and secrete chemokines, cytokines, growth factors, and exosomes that contribute to tissue regeneration (75).

Studies have demonstrated that intravenously administered UC-MSCs can mitigate the development of colitis in mice induced with dextran sodium sulfate (DSS) (78). Intravenously administered UC-MSCs can migrate to sites of intestinal injury, enhance microcirculation, and facilitate tissue repair (126). Transplantation studies using UC-MSCs derived from Kunming mice and humans have shown substantial protective effects against intestinal injury in murine models, significantly reducing inflammation and mortality (129). UC-MSCs alleviate IBD by modulating various immune cell types that interact with their associated cytokines. Previous studies have demonstrated that UC-MSCs inhibit 15-lipoxygenase-1 (15-LOX1) expression in macrophages through the secretion of miR-148b-5p (104) and suppress the phosphorylation of extracellular signal-regulated kinase (ERK) in neutrophils (130), thereby mitigating IBD. In addition to UC-MSCs, clinical trials have shown that BM-MSCs effectively treat moderate-to-severe CD in patients with IBD (77).

MSCs and their exosomes modulate abnormal immune responses and suppress inflammation by targeting various types of immune cells. Furthermore, MSC-Exos alleviate colitis by interacting with the intestinal epithelial cells (IECs) (131). They repair the intestinal epithelial barrier (IEB) (132), decrease colonic fibrosis (133), and reduce oxidative stress. Additionally, MSC-Exos prevent IEC apoptosis and promote the regeneration of intestinal stem and epithelial cells in UC, thereby mitigating colitis (78).

## 6 Impact of IBD on TR-MSCs

Multipotent mesenchymal progenitor cells reside in the lamina propria of both healthy and IBD human colonic mucosa. These cells were identified by their expression of stem cell marker Octamer-Binding Transcription Factor 4 (Oct4) and mesenchymal lineage marker Gremlin 1 (Grem1) (134). In normal gut mucosal tissues, TR-MSCs are recognized as progenitors of lamina propria fibroblasts and myofibroblasts (135) and play a pivotal role in modulating immune responses, facilitating tissue repair, and maintaining intestinal homeostasis (136). Studies have demonstrated that gut mucosal TR-MSCs significantly increase in number in UC but only slightly in CD. Additionally, the progenitor functions of TR-MSCs are differentially impaired in CD and UC.

MSCs isolated from UC tissues maintain their ability to proliferate but lose their adipogenic capacity and show reduced osteogenic differentiation. This loss may have resulted from the local expansion of TR-MSCs in the UC mucosa, leading to the depletion of their regenerative properties. Both regular and UC-derived MSCs that express Oct4 retain clonogenicity and are partially capable of differentiation (137, 138). However, in CD, the number of cells coexpressing Grem1 and Oct4 *in situ* is decreased, and their self-renewal and pluripotency are significantly compromised. Increased inflammation in CD may cause senescence or apoptosis of TR-MSCs (139, 140). The colonic mucosa of patients with UC not only had a significantly higher number of TR-MSCs but also showed active proliferation, as indicated by increased Ki67 expression. Conversely, TR-MSCs from normal and CD colonic mucosae rarely expressed Ki67. These findings highlight the distinct pathological differences between patients with CD and UC. However, the precise consequences of TR-MSCs damage in CD and UC are poorly understood (134).

## 7 Regulatory effects of MSC on the immune microenvironment in IBD

**Figure 1:**
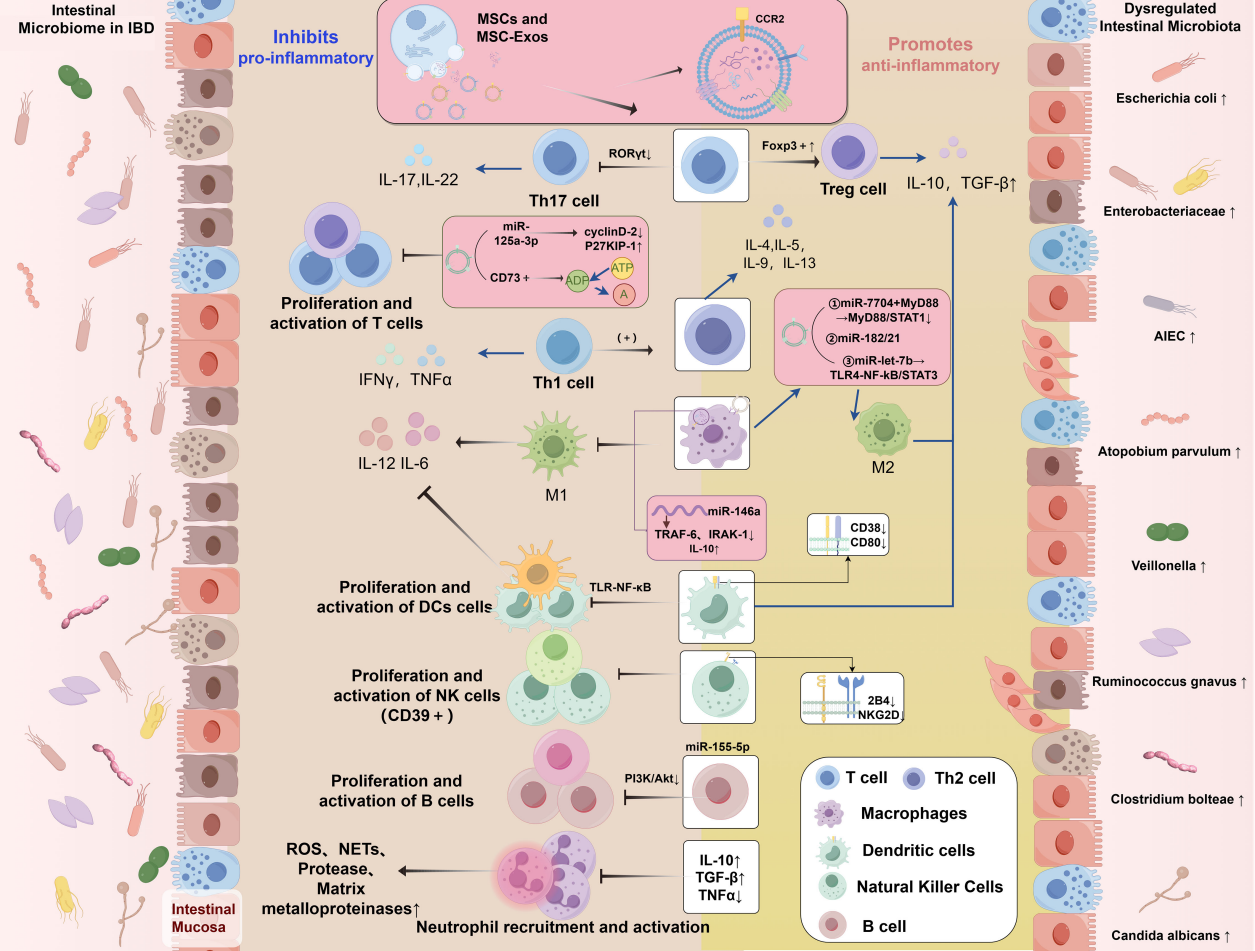
Dysregulation of the intestinal microbiome in IBD and the regulatory role of MSCs in modulating the immune microenvironment of IBD. IBD, inflammatory bowel disease; MSCs, mesenchymal stromal/stem cells; MSC-Exos, mesenchymal stromal/stem cell-derived exosomes; miRNAs, microRNAs; IL, interleukin; TNF-α, tumor necrosis factor alpha; IFN-γ, interferon-gamma; TGF-β, transforming growth factor-beta; ROS, reactive oxygen species; NETs, neutrophil extracellular traps; CCR2, Chemokine (C-C motif) Receptor 2; P27KIP-1, Cyclin-dependent Kinase Inhibitor 1B; CD, Cluster of Differentiation; RORγt, Retinoic acid-related Orphan Receptor gamma t; Foxp3, Forkhead box P3; A, Adenosine; ADP, Adenosine Diphosphate; ATP, Adenosine Triphosphate; MYD88, Myeloid Differentiation Primary Response Gene 88; STAT, Signal Transducer and Activator of Transcription; NF-κB, Nuclear Factor kappa-B; TRAF6, Tumor necrosis factor Receptor Associated Factor 6; IRAK1, Interleukin 1 Receptor Associated Kinase 1; TLR, Toll-like receptor; NKG2D, NK group 2 member D; PI3K, Phosphatidylinositol 3-kinase; AKT, protein kinase B; M1, Classically activated macrophages; M2, Alternatively activated macrophages; Tregs, regulatory T cells; Th, T Helper; DCs, dendritic cells; AIEC, Adherent Invasive Escherichia coli.

MSCs and MSC-Exos have been demonstrated to modulate inflammatory response by targeting various immune cells, including macrophages (133), natural killer (NK) cells, T lymphocytes, B lymphocytes (141), dendritic cells (DCs), and neutrophils (142, 143). Furthermore, MSC-Exos can regulate aberrant immune responses and modulate DNA methylation levels by delivering bioactive substances such as cytokines (78), thereby altering the phenotype and function of immune cells and inhibiting inflammatory responses (**Figure 1**).

### 7.1 Promoting macrophage polarization to the alternatively activated macrophage phenotype

Pro-inflammatory factors stimulate macrophages to migrate to sites of inflammation, where they undergo polarization into distinct phenotypes regulated by chemokines and inflammatory mediators (144). Classically activated macrophages (M1) secrete pro-inflammatory cytokines and Th1 chemokines, which are involved in antigen presentation, T-cell activation, and induction of adaptive immune responses (145). Alternatively activated macrophages (M2) secrete anti-inflammatory cytokines that downregulate the immune response and suppress inflammation (146). An imbalance between M1 and M2 macrophages can lead to persistent inflammation, impeding the normal repair process and causing tissue damage (147). Abnormal macrophage polarization has been reported to disrupt immune regulation in the intestinal mucosa, thereby contributing to intestinal inflammation and the pathogenesis of IBD (148–150). Studies have shown that MSC-Exos regulate macrophage polarization toward the M2 phenotype (133), reduce the M1/M2 ratio, downregulate the expression of pro-inflammatory factors, such as IL-6 and IL-12, and decrease macrophage infiltration in colon tissue (151). Mao et al. found that exosomes derived from human umbilical cord mesenchymal stem stromal/cells (hUC-MSCs) inhibit the expression of IL-7 in macrophages and reduce the inflammatory response, thereby alleviating DSS-induced colitis in mice (152).

TSG-6 is a well-established anti-inflammatory mediator whose upregulation facilitates the conversion of macrophages from the pro-inflammatory M1 phenotype to the anti-inflammatory, tissue-repairing M2 phenotype, a critical process for both suppressing inflammation and promoting tissue repair (153, 154). Studies have demonstrated that human AT-MSCs produce TSG-6, which plays a significant role in alleviating DSS-induced colitis in mice by modulating the immune cell composition within the colon (155). Human mesenchymal stem cells (hMSCs) have been shown to secrete TSG-6 in response to inflammatory stimuli. *In vitro* experiments reveal that BM-MSCs, in conjunction with TSG-6, downregulate the Toll-like receptors (TLR) 2/myeloid differentiation primary response protein 88 (MyD88)/nuclear factor-κB (NF-κB) signaling pathway and reduce the production of pro-inflammatory cytokines such as IL-1β, IL-6, and TNF-α (89). Furthermore, the therapeutic efficacy of hMSCs directly correlates with the mRNA expression levels of TSG-6 (156). A recent study involving AT-MSCs demonstrated that MSC-Exos carrying TSG-6 alleviated inflammation in an IBD mouse model by enhancing colonic regulatory T cells (Tregs) and promoting macrophage polarization from the M1 to the M2 phenotype (87). Additionally, MSC-Exos may amplify TSG-6 signaling by upregulating regulatory molecules, such as specific miRNAs or proteins, in recipient cells (112). Collectively, these mechanisms contribute to the establishment of a predominantly anti-inflammatory environment.

The C-C motif ligand 2 (CCL2) and its primary receptor, C-C motif chemokine receptor-2 (CCR2), are pivotal for recruiting pro-inflammatory monocytes (157). Enrichment of CCR2 in MSC-Exos facilitates binding to the extracellular ligand CCL2, thereby reducing local CCL2 concentrations, inhibiting its activity, and suppressing the recruitment and activation of peripheral monocytes and macrophages (157). CCL2 attracts monocytes and macrophages to inflammation sites (158, 159). Moreover, CCL2 secreted by MSCs and incorporated into exosomes mediates the differentiation of CCR2 ^+^ macrophages into the reparative M2 phenotype (160). This transition leads to the release of TGF-β, which not only promotes fibrosis progression but also inhibits macrophage polarization into the M1 phenotype and reduces the secretion of pro-inflammatory cytokines (161).

**Figure 2:**
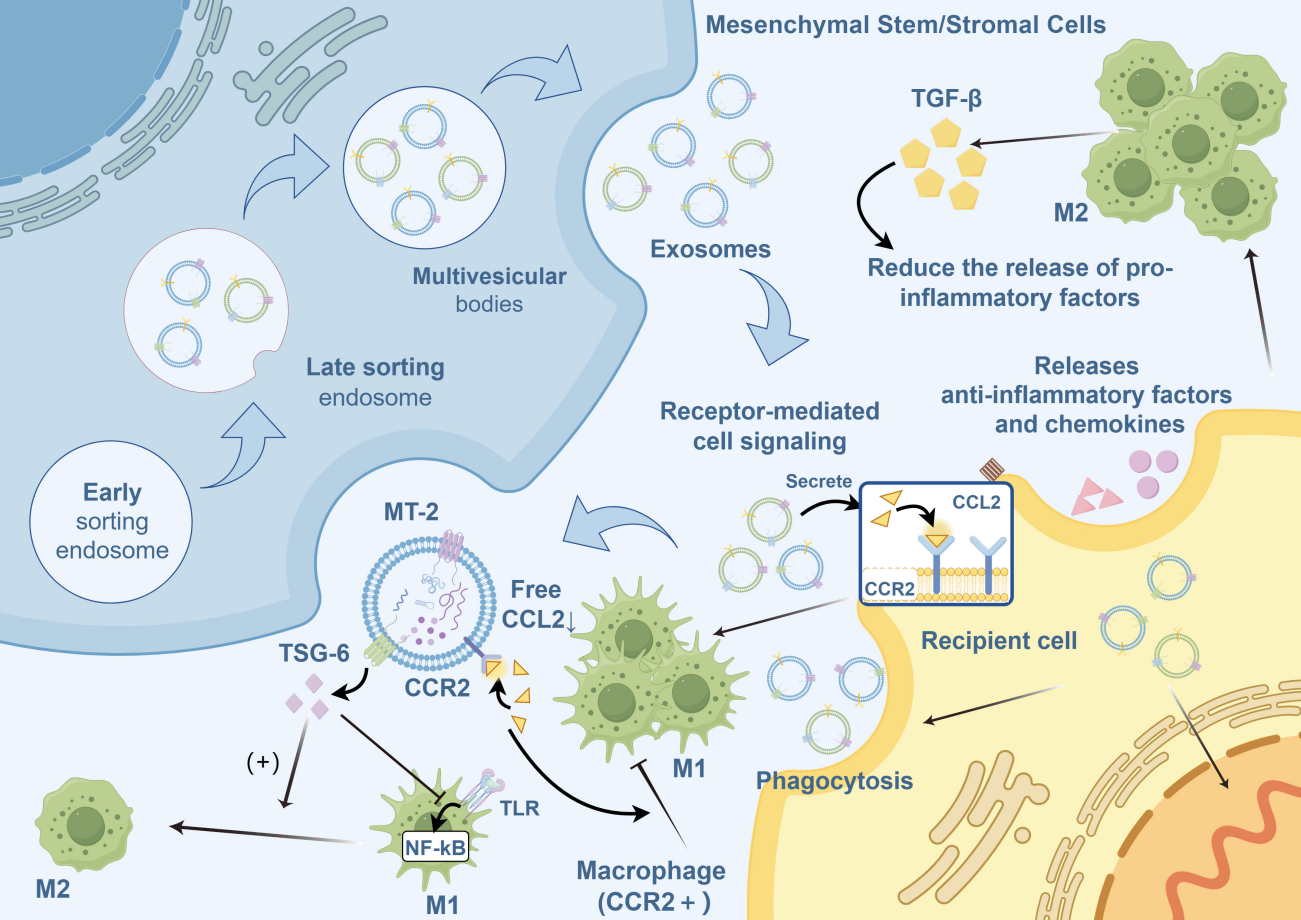
Regulation of macrophage polarization by MSC-Exos.MSC-Exos, mesenchymal stromal/stem cell-derived exosomes; M1, Classically activated macrophages; M2, Alternatively activated macrophages; TGF-β, transforming growth factor-beta; CCL2, C-C motif ligand 2; CCR2, C-C motif chemokine receptor 2; TSG-6, tumor necrosis factor-stimulated gene 6; NF-κB, nuclear factor kappa B; MT, metallothionein; TLR, Toll-like receptor.

MSC-Exos regulate macrophages via the following mechanisms (**Figure 2**):

1. MSC-Exos contain proteins that regulate several biological processes. Notably, metallothionein-2 is essential for inhibiting intestinal inflammation by maintaining the integrity of the intestinal barrier and inducing the polarization of M2 macrophages (133, 162).
2. MSC-Exos target and regulate mRNA expression in macrophages, inhibit the release of pro-inflammatory factors, and promote the secretion of the anti-inflammatory cytokine IL-10 (105, 163, 164).
3. MSC-Exos facilitate macrophage polarization toward the M2 phenotype through TLR. TLR modulates MyD88-dependent TLR signaling through miRNAs (100, 165), particularly via TLR4 (98) or by regulating TLR4, TLR2 (166), and other components of the TLR signaling cascade (167) via miRNA-mediated mechanisms.
4. Macrophages internalize MSC-Exos, leading to the secretion of numerous anti-inflammatory factors and chemokines that regulate the release of inflammatory mediators (168, 169).
5. The binding of CCR2 on MSC-Exos to its ligand CCL2 inhibits the recruitment and activation of monocytes, prevents the polarization of macrophages to the M1 phenotype, and reduces the release of pro-inflammatory factors (170–172).
6. MSC-Exos promote M2-like macrophage polarization by activating AKT/ERK-dependent signaling pathways via a CD73/adenosine-dependent mechanism (173).

### 7.2 Inhibiting NK cells

In the pathogenic microenvironment of IBD, NK cells produce various pro-inflammatory cytokines, such as IFN-γ and TNF-α, which exacerbate intestinal inflammation and mucosal damage (174). NK cells create pores in the target cell membrane by releasing perforin, allowing the release of granzymes to initiate apoptosis, thereby directly killing target cells (175). Consequently, dysregulation or excessive activation of NK cells in the intestine can lead to epithelial cell destruction and disrupt tight junction proteins between the epithelial cell monolayers, increasing intestinal permeability, gut barrier damage, and further inflammation (176). Additionally, NK cells exert their effects by activating the NK group 2 member D (NKG2D) receptor. When the NKG2D receptor is activated, NK cells exhibit enhanced cytotoxicity and release pro-inflammatory cytokines. In IBD, IECs or infiltrating immune cells may express NKG2D ligands, and NK cells recognize and kill these cells via the NKG2D receptor, contributing to intestinal inflammation and tissue damage (177). Furthermore, mutations in Syntaxin-binding protein-2 cause NK cell dysfunction, leading to excessive inflammatory states, including familial hemophagocytic lymphohistiocytosis (HLH) and infantile-onset IBD (178).

A study employing a DSS-induced IBD murine model demonstrated that treatment with human gingiva-derived MSCs markedly suppressed the frequency of splenic NK 1.1^+^, CD11b^+^, and CD4^+^ T cells (179). Although studies directly validating the interactions between MSCs and NK cells within the IBD intestinal microenvironment remain scarce, mass spectrometric analysis, along with other *in vitro* and *in vivo* experiments, have shown that MSCs can suppress the pro-inflammatory actions of NK cells. The liver-resident MSC-mediated reduction in NK cell cytotoxicity occurs through the upregulation of human leukocyte antigen (HLA)-C1 production, which inhibits NK cell activity by stimulating the inhibitory killer cell immunoglobulin-like receptor, two immunoglobulin domains, and the long cytoplasmic tail 2/3 (KIR2DL 2/3) (180). MSCs can also suppress NK cell cytotoxicity by secreting factors such as PGE2 and TGF-β, reducing IFN-γ production and alleviating the inflammatory response (181). This suppression may occur through direct cell-cell contact or by inducing the generation of Tregs, downregulating NK cell activation receptors such as 2B4 (CD244) and NKG2D, and upregulating the expression of inhibitory receptors (182, 183). Furthermore, MSCs influence NK cell proliferation and activation by modulating miRNA expression (184). Collectively, these studies indicated that MSCs may effectively ameliorate IBD by modulating NK cells. However, additional research is needed to provide direct evidence and elucidate the underlying regulatory mechanisms governing this therapeutic approach.

### 7.3 Inhibiting DC maturation and activation

DCs are highly effective antigen-presenting cells characterized by numerous dendritic and pseudopodia-like processes during their maturation. They express and secrete pro-inflammatory cytokines, such as IL-6 and IL-12 (185), and exhibit elevated levels of co-stimulatory molecules, including CD40 and CD80 (105). DCs can exacerbate the inflammatory responses in IBD and impair the integrity of the intestinal mucosa by generating reactive oxygen species (ROS) (186). Reis et al. investigated the mechanisms by which MSC-Exos suppress antigen uptake and differentiation in endothelial cells and demonstrated that MSC-Exos ultimately downregulate the expression of endothelial maturation and activation markers, including CD38, CD80, and CD83 (187). DCs exhibited downregulated expression of pro-inflammatory cytokines, including IL-6, IL-12, and IL-10, alongside upregulated expression of TGF-β. Additionally, MSC-Exos inhibit the maturation and differentiation of DCs by modulating the TLR/NF-κB signaling pathway (188), a key regulator of inflammatory responses, thereby suppressing intestinal inflammation (187). Additionally, MSC-Exos enhance the secretion of anti-inflammatory cytokines, such as TGF-β and IL-10, from CD11c^+^ DCs, which suppress lymphocyte proliferation and promote immune homeostasis (188). Studies have demonstrated that MSC-Exos modulate DC activity by downregulating major histocompatibility complex II and costimulatory molecules on CD11c^+^ DCs in a dose-dependent manner. This modulation induces a hypoactive DC phenotype, thereby impairing antigen presentation and inhibiting T-cell activation and proliferation (189). Additionally, MSC-Exos promote Treg expansion, fostering immune tolerance and suppressing excessive inflammation. These multiple effects underscore the therapeutic potential of MSC-Exos in restoring immune balance and treating IBD (188, 190).

### 7.4 Regulatory effects on T lymphocytes

T cell proliferation and activation play critical roles in the onset and progression of numerous autoimmune diseases. Naïve CD4^+^ T cells (Th0) differentiate into Tregs, Th1, T helper 2 (Th2), and Th17 cells in response to antigen stimulation by intestinal immune cells and cytokine regulation (191). The chronic inflammatory characteristics of IBD are strongly associated with an imbalance between Th1 and Th2 cell subsets in the intestinal microenvironment (191). Similarly, an imbalance between Th17 and Treg cells contributes significantly to a persistent inflammatory state. Therefore, the differentiation trajectory of T-cells is pivotal for preserving intestinal immune homeostasis and modulating inflammatory responses within the gut microenvironment. Restoring the cellular equilibrium has emerged as a pivotal aspect of the therapeutic management of IBD.

Previous studies have demonstrated that when T cells are co-cultured with MSC-Exos, the expression of cyclin D2 is downregulated, whereas that of p27KIP-1 is upregulated (192). This prevents T cells from entering the S phase and inhibits their growth and proliferation. These findings suggest that MSC-Exos exert therapeutic effects in the immune microenvironment of IBD by regulating T cell proliferation and differentiation (193).

Programmed cell death protein 1 (PD-1) is induced following the activation of self-reactive T cell subsets (194). For instance, after CD3/CD28 co-stimulation, PD-1 expression is markedly upregulated. In autoimmune diseases or chronic inflammation, the persistent activation of self-reactive T cells significantly increases PD-1 levels, establishing it as a key regulatory factor in T cell activation and homeostasis (195). MSC-Exos further enhance the immunosuppressive environment by upregulating the expression of specific regulatory molecules. They increase the expression of PD-L1/2 and secrete soluble PD-L1/2 (sPD-L1/2) (195), which binds to PD-1 in self-reactive T cells. This interaction results in various effects: Reduced AKT phosphorylation leads to the upregulation of the downstream inflammatory transcription factor FOXO3, suppressing T cell activation, proliferation, and effector cytokine production (196, 197). Activation of the PD-1 pathway via PD-L1 converts naïve CD4^+^ T cells into Foxp^3+^-induced Tregs, promoting peripheral tolerance (198, 199), including the suppression of IFN-γ, TNF-α, and IL-2 secretion, with the reduction of IL-2 specifically attributed to sPD-L1/2 secreted by MSCs (195, 200). MSC-Exos increase TGF-β expression (201), which further suppresses the proliferation and cytotoxic activity of self-reactive T cells (202, 203).

We identified mechanisms through which MSCs may regulate T cells:

1. **Exonucleases CD73 and CD39 via the Adenosinergic Pathway:** Under certain conditions, particularly during tissue damage, cellular Adenosine Triphosphate (ATP) is released extracellularly. Activated T cells with high CD39 expression convert ATP to Adenosine Monophosphate (AMP), whereas hMSCs and their derived exosomes express high levels of CD73, effectively producing adenosine from AMP. This mechanism enables MSCs to suppress T-cell proliferation via adenosine-mediated signaling pathways (204, 205).
2. **Active Molecules Carried by MSC-Exos:** Molecules such as TGF-β, recombinant indoleamine-2,3-dioxygenase, and miR-125a-3p enable MSC-Exos to inhibit T cell proliferation and activation (142).
3. **Enhancement of Inhibitory Effects on Autoreactive T Lymphocytes:** MSC-Exos potentiate their immunosuppressive effects on autoreactive T cells by enhancing the expression of several regulatory molecules. Notably, they upregulate the expression of PD-L1 and PD-L2 and promote the secretion of their soluble forms (sPD-L1/2).
4. **Regulation of Immune Regulatory Factors:** MSC-Exos inhibit T cell activation and increase the number of Tregs and anti-inflammatory Th2 cells by regulating the production of immune regulatory factors, such as PGE2, HLA-G5, and other membrane-bound molecules (105).

Additionally, MSC-Exos may influence the balance between Th1 and Th2 cells and Tregs and Th17 cells (141). Th1 cells play a critical role in immune response by secreting pro-inflammatory cytokines, including IFN-γ and IL-12, stimulating the activity of macrophages and NK cells (206), enhancing cellular immunity, and accelerating class switching of B lymphocytes. Th2 cells primarily mediate immune responses against extracellular parasites, bacteria, and toxins. Th2 cells secrete cytokines, such as IL-4 and IL-25, promote antibody production, activate eosinophils, and inhibit various macrophage functions. Studies have demonstrated that co-culturing exosomes derived from BM-MSCs with peripheral blood mononuclear cells can promote the transformation from Th1 to Th2 cells, significantly reduce the levels of pro-inflammatory cytokines IL-1β and TNF-α, and increase the level of the anti-inflammatory cytokine TGF-β (207). These findings suggest that MSCs may significantly slow the progression of IBD by modulating T-cell responses.

Th0 cell differentiation into specific T-helper (Th) subsets is predominantly regulated by cytokine concentrations within their microenvironments, with IL-12 serving as a key modulator (141). An increase in IL-12 concentration promotes the differentiation of T cells into Th1 cells (208). Conversely, MSC-Exos induce macrophage polarization to the M2 phenotype and reduce IL-12 levels (209), facilitating the differentiation of T cells into Th2 cells. In a study where MSC-Exos were co-cultured with phytohemagglutinin-activated T cells, MSC-Exos significantly reduced IL-12 expression, inhibited Th1 cell differentiation and proliferation, and promoted T cell differentiation into Th2 cells (141).

In patients with IBD, an elevated Th17/Treg cell ratio in peripheral blood reflects a shift toward a pro-inflammatory state (210). Treg cells maintain immune tolerance by inhibiting effector T cells, secreting anti-inflammatory cytokines such as IL-10 and TGF-β, and are subject to negative feedback regulation. Conversely, Th17 cells express pro-inflammatory factors and promote inflammatory activity in IBD (211). After tail vein injection of MSC-Exos, the ratio of Th17 cells to Treg cells in the mesenteric lymphoid tissue of colitis-induced rats was significantly reduced, and colitis symptoms were markedly improved (141). MSCs suppress the differentiation of T cells into Th17 cells by downregulating the expression of retinoic acid receptor-related orphan receptor gamma t and facilitate the differentiation of T cells into Tregs by upregulating Foxp3^+^ expression. MSCs aid in restoring the immune balance by modulating the Th17/Treg ratio when disrupted. They achieve this by inhibiting the proliferation and activity of Th1 and Th17 cells while promoting Treg-mediated immune tolerance in the mesenteric lymph nodes, ultimately restoring the dynamic balance of Th17/Treg cells and thereby reducing inflammation (105). Additionally, MSC-Exos prevented IBD in mice by restoring mucosal barrier integrity and intestinal immune homeostasis through TSG-6. MSC-Exos also prevent IBD by utilizing TSG-6 to restore the mucosal barrier integrity and maintain intestinal immune homeostasis in mice (212). Additionally, an experiment using a mouse model of IBD demonstrated that UC-MSCs and fetal placenta-derived stem cells enhance Treg activity and suppress Th17-driven inflammation, improving immune homeostasis and promoting colon wall repair (213). These dual actions underscore the therapeutic potential of MSCs in mitigating inflammation and restoring colonic integrity in patients with IBD.

In summary, Th1 and Th17 cells promote inflammation, whereas Th2 and Treg cells exhibit anti-inflammatory effects. However, excess anti-inflammatory cells are not necessarily beneficial. When their levels surpass a certain threshold, their effects may shift from anti-inflammatory to pro-inflammatory. For instance, although Foxp3^+^ Treg cells can suppress inflammation, overactivated Treg cells may further damage the intestinal mucosa (213). Nevertheless, MSCs may contribute to the treatment of IBD by modulating the balance between pro-inflammatory and anti-inflammatory cells.

### 7.5 Regulatory effects on B lymphocytes

B-cells play a critical role in maintaining immune homeostasis on mucosal surfaces, including the gastrointestinal tract. Regulatory B cells (Bregs) have functions similar to those of Tregs. Bregs inhibit autoreactive B cells through cell contact, secreting TGF-β and IL-10, and expressing inhibitory molecules. They suppress Th1 cell responses and Th17 cell differentiation (214), inhibit the secretion of pro-inflammatory cytokines, and promote Treg differentiation (215).

In IBD, B cell abnormalities, such as lymphoplasmacytic infiltration and the production of antimicrobial antibodies with pathological IgG-rich profiles, have been observed (63). In patients with UC, the mucosal B cell compartment undergoes significant perturbation. These include an expansion of naïve B cells and IgG^+^ plasma cells, reduced diversity and maturation, and the emergence of an autoreactive plasma cell clone targeting integrin αvβ6 in inflamed intestinal tissues. This disruption is likely due to chronic antigen-mediated overstimulation of gut follicular B cells within a pro-inflammatory environment, which impairs proper germinal center maturation of IgG^+^ and IgA^+^ plasma cells and may promote extrafollicular development and expansion of autoreactive clones. The IgG-rich B cell response further amplifies local inflammatory cascades by recruiting inflammatory monocytes and Th17 cells via Fcγ receptor (FcγR)-dependent mechanisms. Additionally, it increases the sensitivity of the intestinal mucosa to microbes, diet, and self-antigens (216, 217). Stenotic CD has been linked to selective increases in the number of B and IgG plasma cells (218). Moreover, the proportion of Bregs among total B cells was significantly higher in patients than in healthy controls. Following infliximab treatment, a notable increase was observed in the number of peripheral blood Bregs, correlating with disease remission (219). Conversely, active UC is characterized by a failure in the regulatory control of the B cell compartment, leading to decreased serum levels of IL-10 (220).

In a 2,4,6-trinitrobenzenesulfonic acid (TNBS)-induced colitis mouse model, the intraperitoneal injection of hUC-MSCs resulted in the localization of these labeled cells to the inflamed regions of the colon. This treatment upregulated CD5^+^ B cells in the splenic and mesenteric lymph nodes lymphocytes. Following adoptive transfer, CD5^+^ B cells, primarily found in the peritoneal lavage, ameliorate TNBS-induced colitis by restoring the balance among Treg, Th1, and Th17 cells. Furthermore, hUC-MSCs increase the number of CD5^+^ Breg cells, which in turn suppress T cell proliferation and promote IL-10 production to protect against experimental colitis (221). Another study demonstrated that human bone marrow MSCs can induce a novel Breg cell population characterized by CD23 and CD43 markers. This subset upregulated IL-10 expression and significantly inhibited the secretion of inflammatory cytokines by T cells, thereby improving TNBS-induced colitis in mice (222). Moreover, an *in vitro* study showed that BM-MSCs induced CD1d^+^CD5^+^ Breg cells to produce IL-10, fostering an immunosuppressive environment. This process was linked to stromal-derived factor-1α (SDF-1α) and its receptor C-X-C Motif Chemokine Receptor 7 (CXCR7) (223). These results suggest that MSCs ameliorate IBD by regulating B and Breg cells.

Previous studies have shown that MSC-Exos induce B lymphocytes to downregulate the phosphoinositide 3-kinase (PI3K)/protein kinase B (AKT) signaling pathway via miR-155-5p. The PI3K/AKT pathway is essential for various cellular functions, including growth, survival, and metabolism. By targeting this pathway, MSC-Exos effectively inhibited the proliferation of B cells and impaired their ability to respond to antigens, thereby modulating immune responses (142). Real-time PCR analysis confirmed that the expression of genes important for B-cell immune regulation, such as C-X-C motif chemokine ligand 8 and marginal zone B and B1, was upregulated following MSC-Exos treatment (142). In a study using mouse models of collagen-induced arthritis and delayed-type hypersensitivity, MSC-Exos demonstrated dose-dependent anti-inflammatory effects by inhibiting B cell maturation and inducing Bregs within the lymph nodes (142). These findings suggest that MSCs have similar therapeutic effects in IBD. Overall, further rigorous investigations are needed to validate the therapeutic potential of targeting B cells (including Bregs) and develop effective biologics for IBD treatment. Overall, further rigorous investigations are needed to validate the therapeutic potential of targeting B cells (including Bregs) and develop effective biologics for IBD treatment.

### 7.6 Regulatory effects on neutrophil

Neutrophils are one of the earliest cells to develop during inflammation. They eliminate microorganisms through phagocytosis and detect pathogen-associated and damage-associated molecular patterns (224). Neutrophils recruit other immune cells to the inflammatory sites by secreting cytokines and regulating the immune system. However, excessive recruitment may intensify the inflammatory responses and exacerbate inflammatory injury (225). In the immune microenvironment of IBD, activated neutrophils play a crucial role in the pathogenesis of IBD by producing proinflammatory ROS, neutrophil extracellular traps (NETs), and proteolytic enzymes such as neutrophil elastase, myeloperoxidase, and matrix metalloproteinases (226). These substances exacerbate the inflammatory milieu. Experimental studies have demonstrated that the immunomodulatory effects of neutrophils in IBD involve leukocyte activation, regulation of the innate immune response, and modulation of the responses to oxidative stress. The crosstalk between neutrophils, intestinal inflammation, and microbiota is also crucial for the inflammatory process of IBD (227). Consistently, mucosal inflammation in mice with DSS-induced IBD was significantly ameliorated by inhibiting neutrophil-related immune responses, such as proinflammatory mediators and NET formation (228). MSCs promote the production of IL-10 while simultaneously reducing neutrophil recruitment and aggregation in various experimental models (143). However, evidence regarding the direct regulation of neutrophils by MSCs is limited. Therefore, the underlying mechanisms by which MSCs regulate neutrophils within host-microbiota interactions in IBD under both homeostatic and inflammatory conditions remain to be elucidated.

### 7.7 Regulatory effects on myeloid-derived suppressor cells

Myeloid-derived suppressor cells (MDSCs) represent a distinct subset of immunosuppressive leukocytes that typically emerge through the differentiation of myeloid progenitor cells. These cells are subdivided into two main subtypes: granulocyte-like MDSCs (G-MDSCs) and monocyte-like MDSCs (M-MDSCs). Their differential differentiation may be regulated by various cytokines. In humans, M-MDSCs are predominantly characterized as CD11b^+^CD14^+^CD33^+^HLA-DR^low/-^, whereas G-MDSCs are defined as CD11b^+^CD15^+^HLA-DR^low^CD66b^+^. In mouse models, the corresponding phenotypes are CD11b^+^Ly6C^+^ for M-MDSCs and CD11b^+^Ly6G^+^Ly6C^low^ for G-MDSCs (229). M-MDSCs commonly arise in chronic inflammation, neoplasms, and autoimmune disorders, where they contribute significantly to sustaining immune tolerance and facilitating immune evasion. They primarily mediate immunosuppression by producing key immune-related molecules, such as nitric oxide, IL-10, arginase-1, and inducible nitric oxide synthase, and by modulating T cell activity, thereby playing a pivotal role in regulating immune tolerance and suppressing antitumor responses (230). Although immunosuppression is a hallmark of MDSCs, this property overlaps with the immunomodulatory functions of conventional myeloid cells during inflammation (229).

The peripheral blood of patients with IBD exhibits an increased frequency of cells characterized by the CD14^+^ HLA-DR^-^/^low^ phenotype, indicative of an expanded subset of MDSCs (231). In an IBD model induced by hemagglutinin (HA)-specific CD8^+^ T cells, a cell subset defined as CD31^+^ CD11b^+^ Gr1^+^ Ly-6C^+^ is markedly induced, concurrently conferring tolerance to small intestinal and colonic inflammation (232). Moreover, studies have demonstrated that the repeated transfer of these antigen-specific T cells results in an increased frequency of MDSCs expressing CD11b (Gr-1), along with elevated levels of nitric oxide synthase 2 and arginase in the spleen and intestines of VILLIN-HA mice. MDSCs exert direct immunomodulatory effects by suppressing CD8 ^+^ T cell proliferation through a nitric oxide-dependent mechanism that induces T cell apoptosis (231).

Studies on gliomas and myofibroblasts have revealed that MSCs upregulate CD73 expression in MDSCs via exosomal miR-21, thereby augmenting adenosine synthesis and suppressing the functions of various immune cells (101, 233). Moreover, MSC-Exos promote the differentiation of bone marrow cells into immunosuppressive M2-polarized macrophages in breast cancer (234).

In a DSS-induced IBD model, after three cycles of administration, Muc1 knockout (KO) mice—with deletion confined to the hematopoietic compartment—exhibited a significant increase in CD11b^+^Ly-6Ct (Gr1^+^) cells concomitant with a marked reduction in colonic inflammatory lesions. Poh et al. further demonstrated that the absence of Muc l in the bone marrow of Muc l KO mice results in an expansion of CD11b^+^Gr1^+^ MDSCs with immunosuppressive properties, thereby promoting tumor progression. In chimeric mice, hematopoietic Mucl deletion led to enhanced cellular expansion and alleviation of chronic colonic inflammation compared with wild-type (WT) controls. Moreover, during colitis, a transient inflammatory expansion of CD11b^+^Gr1^+^ MDSCs creates a tumorigenic microenvironment that contributes to the progression of colitis-associated colon cancer (CAC). Notably, blockade of these cells in the murine mucosa reduces tumor incidence and significantly upregulates CCL2 and CCR2 expression in CAC (235), suggesting that although CD11b^+^Gr1^+^ MDSCs effectively attenuate inflammation and ameliorate IBD, they may concurrently possess tumorigenic potential. Several studies have demonstrated that MSCs enhance mucin-1 (MUC1) expression (78, 236, 237), suggesting that MSCs may also modulate MDSC functionality through MUC1.

However, given the heterogeneity of MDSCs and their divergent immunomodulatory roles, ranging from suppression to promotion, across different stages of autoimmune diseases (238, 239), as well as the distinct regulatory effects of MSCs and their secreted exosomes on MDSCs, further investigation is warranted to elucidate the complex mechanisms underlying the MSC-mediated regulation of MDSCs within the IBD immune microenvironment.

## 8 Role of MSCs in the repair microenvironment of IBD

**Figure 3:**
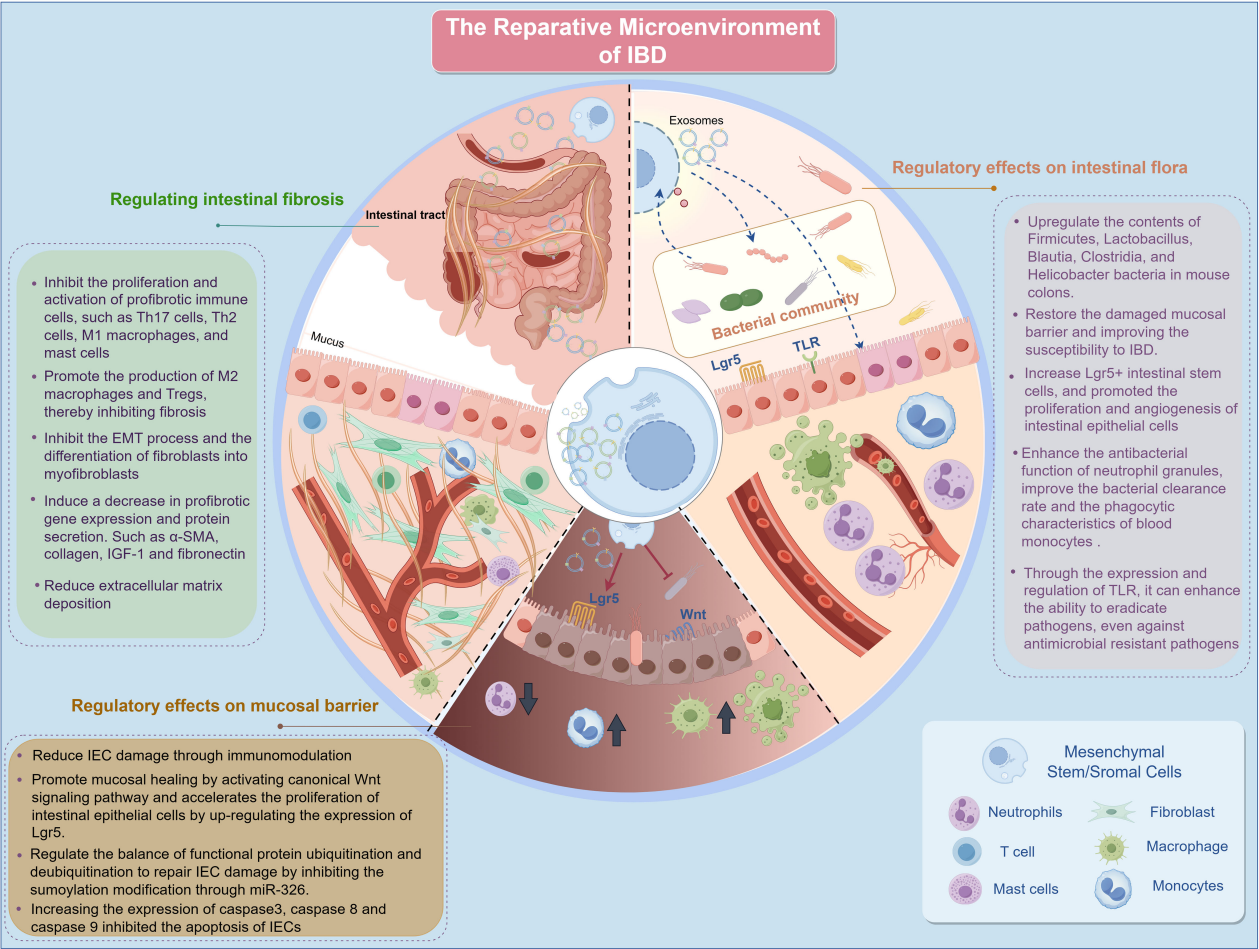
Repair microenvironment of IBD. IBD, inflammatory bowel disease; IEC, intestinal epithelial cells; M1, Classically activated macrophages; M2, Alternatively activated macrophages; EMT, epithelial-to-mesenchymal transition; α-SMA, alpha-smooth muscle actin Lgr5, leucine-rich repeat-containing G-protein-coupled receptor 5; IGF-1, insulin-like growth factor 1; Th, T Helper; TLR, Toll-like receptor; miRNAs, microRNAs.

In the reparative microenvironment of IBD, MSCs play a significant role and have broad therapeutic potential. MSCs can alter the composition of the gut microbiota, thereby restoring the gut microbiota balance and metabolism. Additionally, MSCs promote the repair of damaged mucosal barriers through multiple mechanisms. MSCs have been observed to modulate intestinal fibrosis, a key complication of IBD, via various pathways (**Figure 3**).

### 8.1 Regulatory effects on intestinal flora

Commensal bacteria affect host health and immunity through various mechanisms that promote intestinal immune system development (240). Establishing a healthy gut microbiota during the early life stages is crucial for the optimal functioning of the intestinal immune system. Disrupting this process can lead to an increased incidence and severity of certain intestinal diseases, including UC, CD, and colorectal cancer (241).

IBD is significantly associated with decreased gut microbial diversity (gut microbiota dysbiosis), resulting from imbalances between commensal and potentially pathogenic microorganisms. Numerous studies have consistently demonstrated that the gut microbiota composition in patients with IBD differs significantly from that in healthy individuals. These differences are particularly evident in the microbial diversity and relative abundances of specific bacterial taxa. In patients with CD, a positive correlation exists between the proportions of caudoviruses and Enterobacteriaceae, including *Pasteurella*, all of which are increased (242). The proportion of thick-walled fungi, particularly *Faecalibacterium prausnitzii*, is often reduced in the stool and urine of patients with CD (243). Proteobacteria such as Enterobacteriaceae, including *Escherichia coli*, are generally more abundant in patients with IBD than in healthy individuals. Fungal dysbiosis in IBD is associated with an increased proportion of Basidiomycetes and Ascomycetes, a decreased proportion of *Saccharomyces cerevisiae*, and an increased abundance of *Candida albicans* (244, 245). In addition to the quantity and species of intestinal flora associated with IBD, changes in intestinal flora composition, leading to alterations in their metabolites, are also associated with IBD. Previous studies identified a reduction in amino acid biosynthesis and carbohydrate metabolism pathways within the IBD-associated microbiome. This reduction may contribute to enhanced microbial nutrient uptake, increased virulence, and the activation of secretory pathways. Additionally, genes associated with oxidative stress, including glutathione and sulfate transport genes, are upregulated in colitis (246). Specific gut microbiota and microbial components can activate TLR2, triggering downstream signaling pathways such as MyD88 and PI3K. This activation stimulates IL-10 production by B cells. IL-10-producing B cells interact with T cells to mitigate T cell-mediated chronic colitis (241).

Recently, MSCs have been found to recalibrate immune homeostasis and contribute to the treatment and improvement of various diseases. UC-MSCs alter the composition of the gut microbiota and enhance its diversity, resulting in the restoration of the gut microbiota, metabolism, and repair of the damaged mucosal barrier. Additionally, changes in the composition of the intestinal flora may influence susceptibility to DSS-induced colitis. A 16S ribosomal RNA (rRNA) gene analysis revealed a significant increase in the relative abundance of probiotics, including *Firmicutes*, *Cyanobacteria*, *Roseobacteria*, *Helicobacter pylori*, and *Lactobacillus*, in DSS-induced CD mice treated with MSCs (78). Soonthararak et al. demonstrated that induced pluripotent stem cells, which were initially functionally equivalent to adipose-derived MSCs, triggered an increase in leucine-rich repeat-containing G-protein-coupled receptor 5 (Lgr5)^+^ intestinal stem cells and promoted the proliferation and angiogenesis of IECs, significantly restoring the altered intestinal microbiome in IBD mice (247). Similarly, MSC infusion causes an initial change in the Bacteroidetes/Firmicutes ratio, which maintains intestinal mucosal function and homeostasis (248). TLR2, TLR5, and TLR4 are responsible for recognizing extracellular microorganisms, with TLR4 playing a pivotal role in the primary line of defense against potentially pathogenic bacteria. MSCs can eradicate pathogens, stimulate anti-inflammatory responses, and combat antimicrobial-resistant pathogens by expressing and regulating TLRs (249, 250). Furthermore, studies on other diseases have indicated that BM-MSCs enhance the antimicrobial function of neutrophil granules (251) and improve bacterial clearance and phagocytic properties of blood monocytes (252). Other antimicrobial secretions of MSCs known to directly inhibit bacterial growth or kill bacteria include cathelicidin, lipocalin-2, elastin, and β-defensin-2 (248). Additionally, intestinal endothelial MSCs can undergo a switch to secretory endothelial differentiation in the presence of pathogens, promoting the rapid proliferation and differentiation of goblet and Paneth cells (253). These cells synthesize a range of proteins, including phagocytoproteins, mucins, trefoil factor 3, lysozymes, and defensins, which facilitate bacterial clearance (254).

In addition to gut microbiota regulation by MSCs, studies indicate that the gut microbiota also regulates MSC function. Xiao et al. found that microbiota alters the differentiation potential of BM-MSCs and enhances their immunomodulatory capacities. The microbiota in specific pathogen-free (SPF) mice significantly alters the properties of BM-MSCs compared to those derived from germ-free (GF) mice. Conversely, colonization of SPF microbiota by BM-MSCs from GF mice normalized the proliferation and differentiation capacities of GF mouse-derived BM-MSCs. Moreover, the investigators found that BM-MSCs require normal microbiota to maintain their immunomodulatory properties by modulating the activity of activated T cells. Additionally, GF-derived BM-MSCs lost the ability to ameliorate the disease phenotype in mice with experimental colitis induced by DSS. Restoring gut microbiota diversity protects BM-MSCs from premature aging-related degeneration and loss of cellular growth and division capacity (senescence) (255). Other studies have demonstrated that colonization with normal gut microbiota can restore the functional capacity of BM-MSCs to activate hematopoiesis and erythropoiesis (256).

In addition to MSCs, MSC-Exos play a role in regulating gut microbiota. Human umbilical cord MSC-derived exosomes (HucMSC-Exo) (257) and human fetoplacenta MSC-derived exosomes have been shown to enhance colon wall architecture in mice, thereby preserving colonic integrity (213). This preservation decreases the abundance of proinflammatory gut bacteria. Additionally, enhancing the intestinal barrier may facilitate the proliferation of beneficial bacteria, thereby reducing the incidence of colitis.

### 8.2 Repair of the intestinal barrier

The IEB consists of mechanical, chemical, biological, and immune components that collectively form crucial defense mechanisms against external pathogen invasion. The mechanical barrier, a fundamental component, consists of a physical barrier formed by the tight junctions of IECs. In IBD, imbalances and disruptions in mucosal immunity, surface mucus, intestinal flora, oxidative stress, and other factors compromise IEB, leading to IEC necrosis, apoptosis, and increased intestinal permeability. With advancements in MSC research on the repair of various organs, increasing evidence suggests that MSCs can repair damage to IECs (141). MSCs maintain IEB integrity through several mechanisms. They significantly reduce IEC injury and oxidative stress via immune regulation (258). MSCs also regulate the balance between the ubiquitination and deubiquitination of functional proteins by inhibiting SUMOylation via the high expression of miR-326 in exosomes, thereby repairing IEC damage (259). Additionally, MSCs prevent IEC apoptosis by upregulating the expression of caspase-3, caspase-8, and caspase-9 (258).

**Figure 4:**
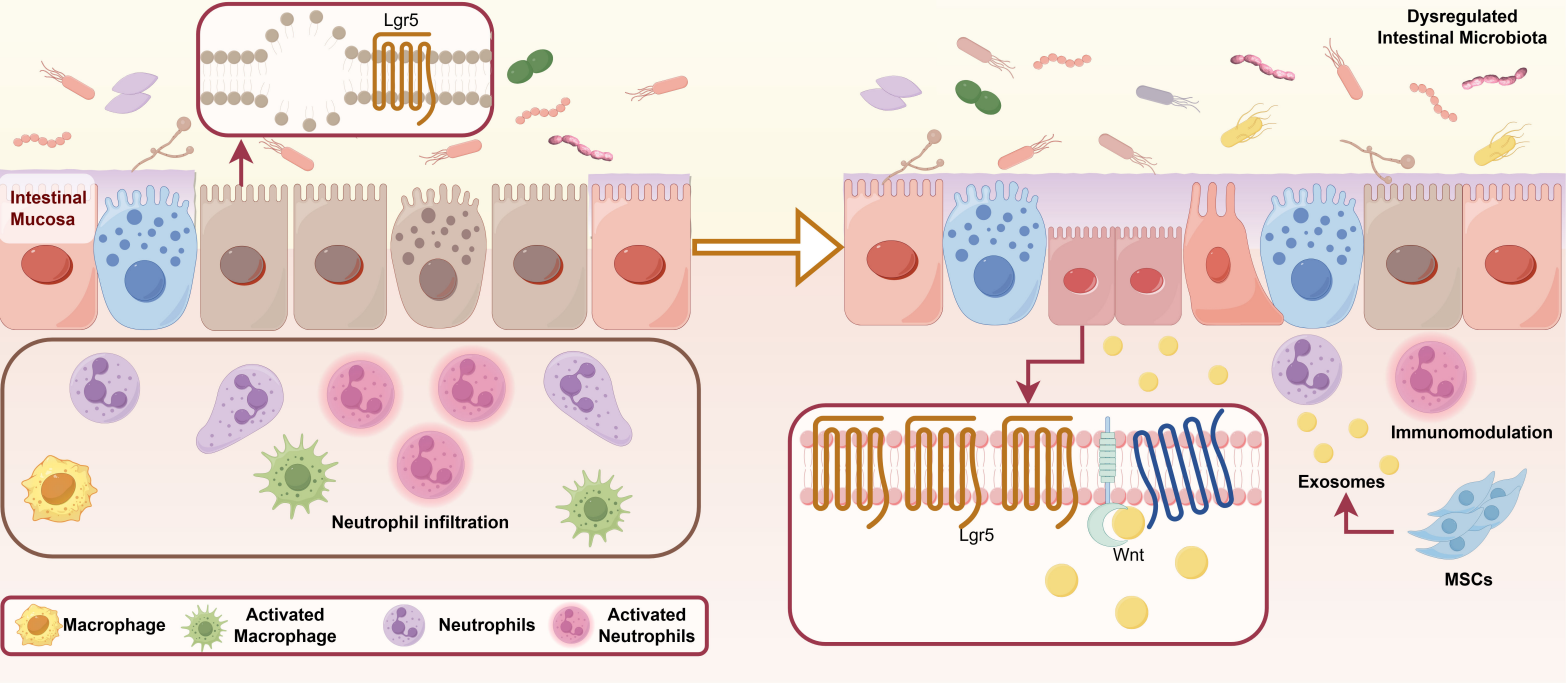
Restoration of the damaged mucosal barrier by MSCs. The fundamental causes of pathological changes in IBD include imbalances and disorders of mucosal immunity, surface mucus, intestinal bacteria, and other factors, which disrupt the IEB, leading to IEC necrosis, apoptosis, and increased intestinal wall permeability. Additionally, MSC-Exos can significantly reduce IEC damage through immune regulation. MSC-Exos activate the canonical Wnt signaling pathway to promote mucosal healing, which is characterized by reduced histopathological damage, decreased neutrophil infiltration, and upregulation of Lgr5, thereby accelerating the proliferation of IECs. IL-25 enhances the ability of MSCs to induce intestinal epithelial cell regeneration. MSCs alter the composition and diversity of the gut microbiota, thereby restoring gut microbiota balance and metabolism. IBD, inflammatory bowel disease; MSCs, mesenchymal stromal/stem cells; MSC-Exos, mesenchymal stromal/stem cell-derived exosomes; IEB, intestinal epithelial barrier; IEC, intestinal epithelial cells; Lgr5, leucine-rich repeat-containing G-protein-coupled receptor 5; Th, T Helper; IL, interleukin.

Studies on MSC-Exos have demonstrated that HucMSC-Exo can activate the canonical wingless-related integration site (Wnt) signaling pathway to promote mucosal healing. This process is characterized by a reduction in histopathological damage and neutrophil infiltration, upregulation of Lgr5 expression, accelerated proliferation of IECs, and increased sensitivity of intestinal organoids to TNF-α, which facilitates growth and budding (260). Additionally, IL-25 enhances the ability of MSCs to induce IEC regeneration, working synergistically to improve epithelial integrity (261) (**Figure 4**).

### 8.3 Improvement of a critical complication: fibrosis

Currently, research on the role and mechanisms of action of MSCs in intestinal fibrosis is limited. However, existing studies have demonstrated the potential of MSCs in treating intestinal fibrosis, indicating a viable approach for preventing and managing fibrosis. MSCs regulate cellular processes during fibrosis by producing cytokines, growth factors, and EVs, which are associated with their regulatory effects on various immune cells. Because tissue fibrosis inherently involves the participation of various immune cells, it is closely linked to the inflammatory response. Studies have demonstrated that MSCs inhibit the proliferation and activation of various immune cells involved in the promotion of tissue fibrosis, including Th17 and Th2 cells, M1 macrophages, and mast cells. In addition, MSCs promote the differentiation of macrophages into the M2 phenotype and enhance Treg expansion. This dual mechanism ultimately inhibited fibrosis. Additionally, MSCs impede epithelial-to-mesenchymal transition (EMT) and myofibroblast generation (68).

In a mouse model of colitis induced by TNBS, MSCs were genetically engineered to overexpress hypoxia-inducible factor 1-alpha and telomerase (MSC-T-HIF). This genetic modification results in the release of EVs that exhibit potent immunomodulatory activity (EV-MSC-T-HIF) in response to proinflammatory stimuli. EV-MSC-T-HIF not only inhibited the differentiation of TGF-β-treated fibroblasts into myofibroblasts but also induced the repolarization of monocytes from the M1 macrophages to the M2-like macrophage phenotype, thereby reducing the release of inflammatory cytokines (262). This approach significantly attenuated fibrosis progression in a mouse model of colitis and demonstrated that MSCs can exert enhanced immunomodulatory effects via gene editing, serving as a bio-delivery vehicle for precision medicine.

In another study using a colorectal fibrosis model, MSCs inhibited fibrogenesis by promoting ECM turnover and reducing ECM deposition in the intestinal wall. Subsequent studies have demonstrated that MSCs reduce profibrotic gene expression and protein secretion via the release of HGF and TSG-6 (263). This antifibrotic effect extends beyond the gut and has been observed in fibrotic processes in other tissues, including the liver and lungs. Regardless of their tissue origin, MSCs have demonstrated the ability to attenuate fibrotic responses and promote tissue repair (264, 265). Additionally, MSCs can reduce collagen deposition in the intestinal wall by inhibiting TGF-β1 protein expression, thus exerting their anti-fibrotic effect (141).

## 9 Potential regulatory targets of mesenchymal stem cells in the repair microenvironment of IBD

### 9.1 NOD-like receptor protein 3 inflammasome

The NOD-like receptor protein 3 (NLRP3) inflammasome is a pivotal component of the innate immune system, playing a central role in sensing a wide array of stress signals and foreign microorganisms, thereby initiating an inflammatory response. This cytosolic protein complex is ubiquitously distributed across the epithelial cells and various immune cell types, including neutrophils (266), monocytes (267), and lymphocytes (268). The NLRP3 inflammasome consists of three main structural domains: the N-terminal pyrin domain (PYD), the central nucleotide-binding and oligomerization domain (NACHT), and the C-terminal leucine-rich repeat (LRRs) domain. The LRR domain recognizes a broad array of ligands, including endogenous danger signals released during cellular damage and microbial components from pathogens. Furthermore, activation of the NLRP3 inflammasome leads to caspase-1 activation and the subsequent processing of pro-inflammatory cytokines IL-1β and IL-18, which are essential mediators of the inflammatory response (268, 269). A study employing MCC950, a selective NLRP3 inflammasome inhibitor, demonstrated that inhibiting inflammasome activity significantly reduced levels of NLRP3, caspase-1, IL-1β, and IL-18 (270), providing evidence for the central role of NLRP3 inflammasome signaling in regulating inflammation and the expression of these pro-inflammatory proteins.

The NLRP3 inflammasome serves as the body’s primary protective response against infection or injury (271) and plays a critical role in shaping the gut microbiome. Activation of the NLRP3 inflammasome in the intestinal epithelium maintains homeostasis by regulating the commensal flora, eliminating pathogenic bacteria, and promoting defensin synthesis. Previous studies have demonstrated that NLRP3 knockout (NLRP3^-/-^) mice exhibit increased susceptibility to colitis, likely due to alterations in gut microbiota composition and subsequent changes in β-defensin levels. When intestinal epithelial integrity is compromised, particularly in the early stages of the disease, NLRP3 inflammasome activation can facilitate intestinal mucosal repair and regeneration. However, overactivation of the NLRP3 inflammasome leads to excessive production of IL-1β, which, while capable of maintaining intestinal homeostasis, also enhances anti-inflammatory responses by remodeling the intestinal microbiota. This remodeling promotes the induction of Tregs and increases the resistance to experimental colitis. Differential expression of NLRP3 may lead to varied outcomes, potentially due to variations in the genetic backgrounds of mice and humans, differences in gut microbiota composition, the selected colitis model, or the colitis induction method. Alternatively, it may result from the location-specific activation of the NLRP3 inflammasome. Generally, activation of the NLRP3 inflammasome in IBD can exacerbate inflammation, worsen colonic injury, or ameliorate colitis to prevent further damage (269). Andrographolide, a herbal extract, enhances mitochondrial autophagy in macrophages, inhibits the NLRP3 inflammasome, and ameliorates DSS-induced colitis (272). These findings underscore the pivotal involvement of NLRP3 inflammasome in the pathogenesis of IBD.

Moreover, emerging research indicates that the NLRP3 inflammasome exerts regulatory effects on MSCs, thereby influencing the therapeutic efficacy of MSC-based interventions. IL-10 is a key immunomodulatory cytokine that protects against excessive immune responses and plays an essential role in maintaining intestinal homeostasis by regulating the immune balance and preventing chronic inflammation (273). NLRP3 inhibitor MCC950 suppressed NLRP3 expression and significantly reduced IL-10 production by MSCs. Conversely, NLRP3 overexpression enhances IL-10 production and therapeutic efficacy. Furthermore, macrophage function is closely associated with the NLRP3 inflammasome. NLRP3 activation in macrophages requires glycolysis, which is regulated by intracellular metabolic pathways influenced by activated NLRP3 inflammasomes. Interestingly, activated inflammasomes modulate glycolytic pathways, indicating a reciprocal regulatory relationship (274). Further studies have demonstrated that NLRP3 deficiency downregulates glucose transporter protein 1 (GLUT1) expression and glycolytic activity in MSCs, leading to decreased IL-10 production and reduced therapeutic efficacy against DSS-induced colitis. In contrast, GLUT1 upregulation in MSCs deficient in NLRP3 significantly enhanced their therapeutic efficacy by restoring IL-10 production and augmenting their protective role against intestinal inflammation (86). MSC-Exos have been shown to modulate NLRP3 inflammasome activity and attenuate spinal neuroinflammation in interstitial cystitis by inhibiting NLRP3 inflammasome activation and downregulating TLR4/NF-κB signaling pathway activity (236). A single high-dose treatment with hMSC-derived exosomes has been shown to inhibit the activation of the NLRP3 inflammasome following traumatic brain injury, as indicated by reduced levels of NLRP3, CARD-containing osteogenic-associated speck-like protein, activated caspase-1, IL-1β, and IL-18 in the injured brain (275). The regulatory effect of MSC-Exos on NLRP3 inflammasome broadens the potential mechanisms by which MSCs exert therapeutic benefits in IBD. Chronic inflammation in IBD is associated with altered adaptive immune responses, including the Th2 profile in patients with UC and the Th1/Th17 profile in patients with CD. Macrophages and their secreted cytokines play central roles in shaping the disease microenvironment. An *in vitro* study demonstrated that hMSCs negatively regulate the activation of the NLRP3 inflammasome in both human and murine macrophages, leading to reduced caspase-1 activation and subsequent IL-1β release. Further investigation revealed that NLRP3-activated macrophages stimulate hMSCs to upregulate and secrete the anti-apoptotic protein stanniocalcin-1 (STC-1), which, in turn, suppresses macrophage NLRP3 inflammasome activation and reduces ROS production (276). Another study indicated that hMSCs express essential components required for NLRP3 inflammasome assembly. Moreover, the suppressive effects of MSCs on T-cell responses and macrophage activation were enhanced following NLRP3 activation. Under such stimulation, MSCs do not undergo pyroptosis nor produce significant amounts of IL-1β. *In vivo* studies in mice with colitis demonstrated that after NLRP3 stimulation, hMSCs exhibit enhanced protective effects. *In vitro*, NLRP3 inflammasome activation in hUC-MSCs inhibits IL-1β and TNF-α production by M1 macrophages, suppresses T cell proliferation, significantly reduces IFN-γ production by Th1 cells, slightly inhibits IL-4 production by Th2 cells, and promotes IL-10 production by T lymphocytes, without modulating Treg cell activity (277).

In addition to their role in IBD, the therapeutic effects of MSCs and their exosomes in the regulation of NLRP3 inflammasome activation have been extensively studied in inflammatory diseases of the central nervous system (278) and myocarditis (270). MSCs can significantly inhibit the expression of nucleotide-binding oligomerization domain (NOD) 2, NLRP3, caspase-1, IL-1β, and IL-18 mRNA in the left ventricle of coxsackievirus B3-infected mice. MSCs also reduce NOD2 expression, NLRP3 inflammasome activation, and IL-1β secretion in HL-1 cells after infection. Furthermore, the levels of NLRP3, IL-1β, and IL-18 are significantly elevated in spinal cord injury rats, with a marked reversal observed following MSC transplantation. However, additional research is required to elucidate the complex relationship between MSCs and NLRP3 inflammasome activation in patients with IBD.

**Figure 5:**
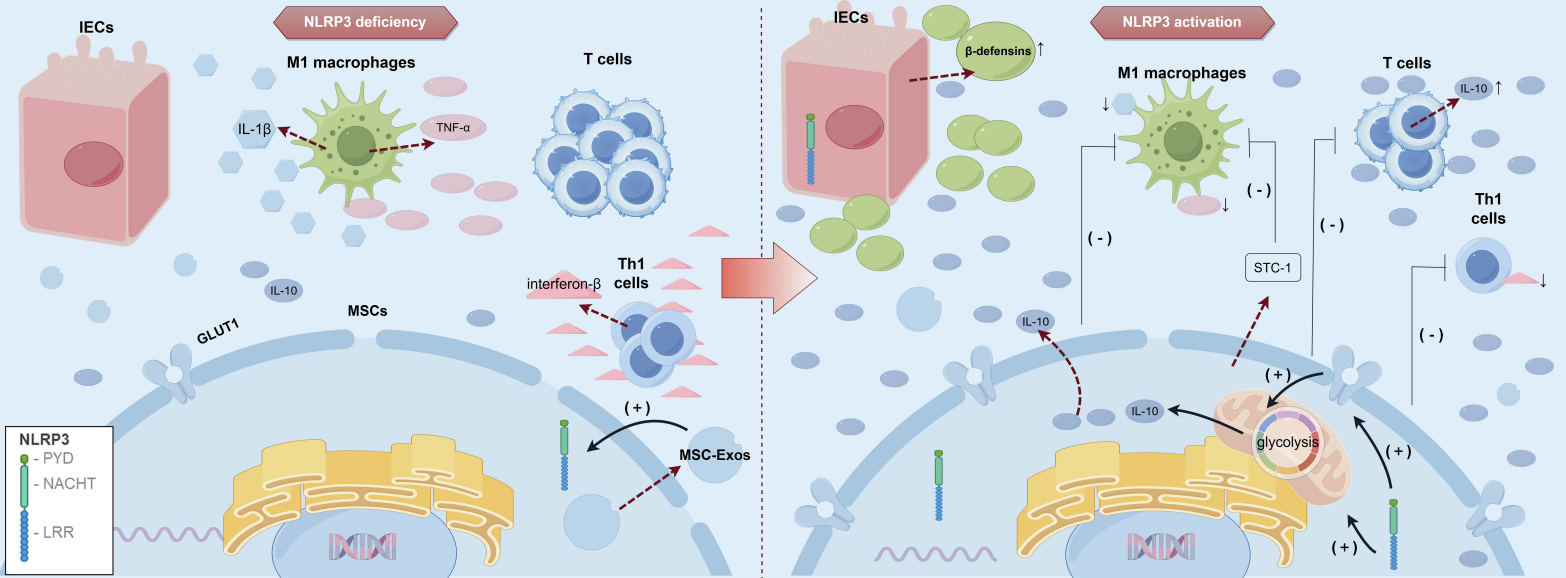
Role of NLRP3 inflammasome activation in MSCs and their immunomodulatory effects. In the intestinal microenvironment of IBD: Activated NLRP3 can stimulate the production of intestinal defensins. Enhanced immunoregulatory ability of MSCs: 1. Up-regulation of IL-10 production by Glut1; 2. Inhibit T cell proliferation, Th1 cell differentiation, Th1 cell function, and promote Th2 cell function; 3, inhibit the activation of M1 macrophages. MSC-Exos also regulate the activity of NLRP3 inflammasome. IBD, inflammatory bowel disease; MSCs, mesenchymal stromal/stem cells; MSC-Exos,mesenchymal stromal/stem cell-derived exosomes; M1, Classically activated macrophages; IEC, intestinal epithelial cells; IL, interleukin; NLRP3, NOD-like receptor protein 3; Th, T Helper; Leucine-Rich Repeat, LRR; Nucleotide-binding and oligomerization domain, NACHT; Pyrin domain, PYD; GLUT1, glucose transporter protein 1; STC-1, stanniocalcin-1.

Overall, MSCs and their exosomes positively modulate the gut immune microenvironment by interacting with the NLRP3 inflammasome, regulating various immune cells (including T cells, Th cells, and macrophages), and exerting significant anti-inflammatory effects on IBD. This represents a promising therapeutic strategy (**Figure 5**).

### 9.2 MUC1

MUC1 is a transmembrane glycoprotein predominantly expressed on the apical surface of epithelial cells within the ductal structures of various organs, where it contributes to mucosal protection and lubrication. Additionally, it has been identified as a tumor-associated antigen that plays a pivotal role in cell signaling and adhesion and is overexpressed in various human adenocarcinomas. Shortly after synthesis, its extracellular domain undergoes proteolytic cleavage within the sea urchin sperm protein, enterokinase, and agrin module, which consists of 120 amino acids. This cleavage results in two subunits, α and β, which specifically recognize each other and bind together through strong non-covalent interactions (279). The peptide backbone of MUC1 is characterized by a variable number of tandem repeat (VNTR) regions, averaging 80–200 repeats, each 20 amino acids in length. In healthy epithelium, MUC1 expression is reduced owing to extensive glycosylation of the VNTR region by long-branched O-chain carbohydrates. However, in most human adenocarcinomas and their precursor lesions, tandem repeats are overexpressed because of extensive hypoglycosylation (280). Furthermore, in cancer-associated MUC1, the carbohydrate side chains become incomplete due to the loss of polarity in epithelial cells, leading to the formation of new carbohydrate structures, such as Thomsen-Friedenreich, Tn, and sialyl-Tn antigens, and exposure of the core peptide (281).

In a DSS-induced model of IBD, Muc1-KO mice restricted to the hematopoietic compartment were significantly healthier than control mice over three drinking cycles. However, after the third cycle, a significant reduction in soluble receptor for advanced glycation end product (sRAGE) levels and a substantial increase in colon tumor burden were observed in KO mice compared to WT mice (235). Furthermore, injected CD4^+^ and CD8^+^ T cells migrated to the colon of MUC1^+^IBD mice in a MUC1-specific manner, whereas MUC1-specific cells did not migrate to the colon in healthy control mice. Therefore, Muc1 deficiency intensifies chronic inflammation in both Th1- and Th2-mediated colitis models (389). Poh et al. demonstrated that deletion of hematopoietic Muc1 in chimeric mice (KO WT) resulted in increased cell expansion and alleviation of chronic colonic inflammation (235).

The pathogenesis of IBD is primarily driven by an inflammatory cascade characterized by the increased production of TNF-α. Elevated TNF-α activity results in MUC1 upregulation. MUC1 expression is regulated by inflammatory cytokines, including IFN-γ and TNF-α (282, 283). Additionally, repeated cycles of inflammation, injury, and regeneration lead to the loss of cell polarity and increased expression of the low-glycosylated form of MUC1 (abnormal form) (280). MUC1 expression and localization exhibit different patterns under different pathological changes in the colonic mucosa (284).

The abnormal form of MUC1 is associated with impaired barrier function and increased susceptibility to further inflammatory insults. This suggests that MUC1 plays a protective role in the intestinal epithelium during IBD. Dysregulation of this aberrant form of MUC1 accelerates IBD development and contributes to colon cancer progression by amplifying inflammatory responses in the gut (285). This is attributed to the ability of abnormally expressed and hypoglycosylated MUC1 to attract innate immune cells and promote acute inflammation, which gradually progresses to chronic inflammation (286). These findings may be explained by the abnormal glycosylation of MUC1 mucin in tumor cells, which is recognized by immature human myeloid DCs as a chemoattractant (through its polypeptide core) and as a signal for maturation and activation. Additionally, MUC1 promotes cellular transformation, at least in part, by inhibiting glycogen synthase kinase 3 beta-mediated phosphorylation and subsequent degradation of β-catenin. CD4^+^ and CD8^+^ T cells specifically recognize the aberrant form of MUC1. Overall, the presence of MUC1 has a profound impact on inflammation onset and severity and colon cancer progression in IBD mouse models.

Ferroptosis, a form of regulated cell death characterized by iron accumulation and induction of oxidative stress, has emerged as a significant contributor to the development of IBD (287). Immune pathways in the gut may modulate signals associated with cell death, including ferroptosis and apoptosis, leading to the loss of IECs, which further amplifies inflammatory responses and disrupts the intestinal barrier. Notably, ferrostatin-1 can reduce the severity of TNBS-induced colitis.

**Figure 6:**
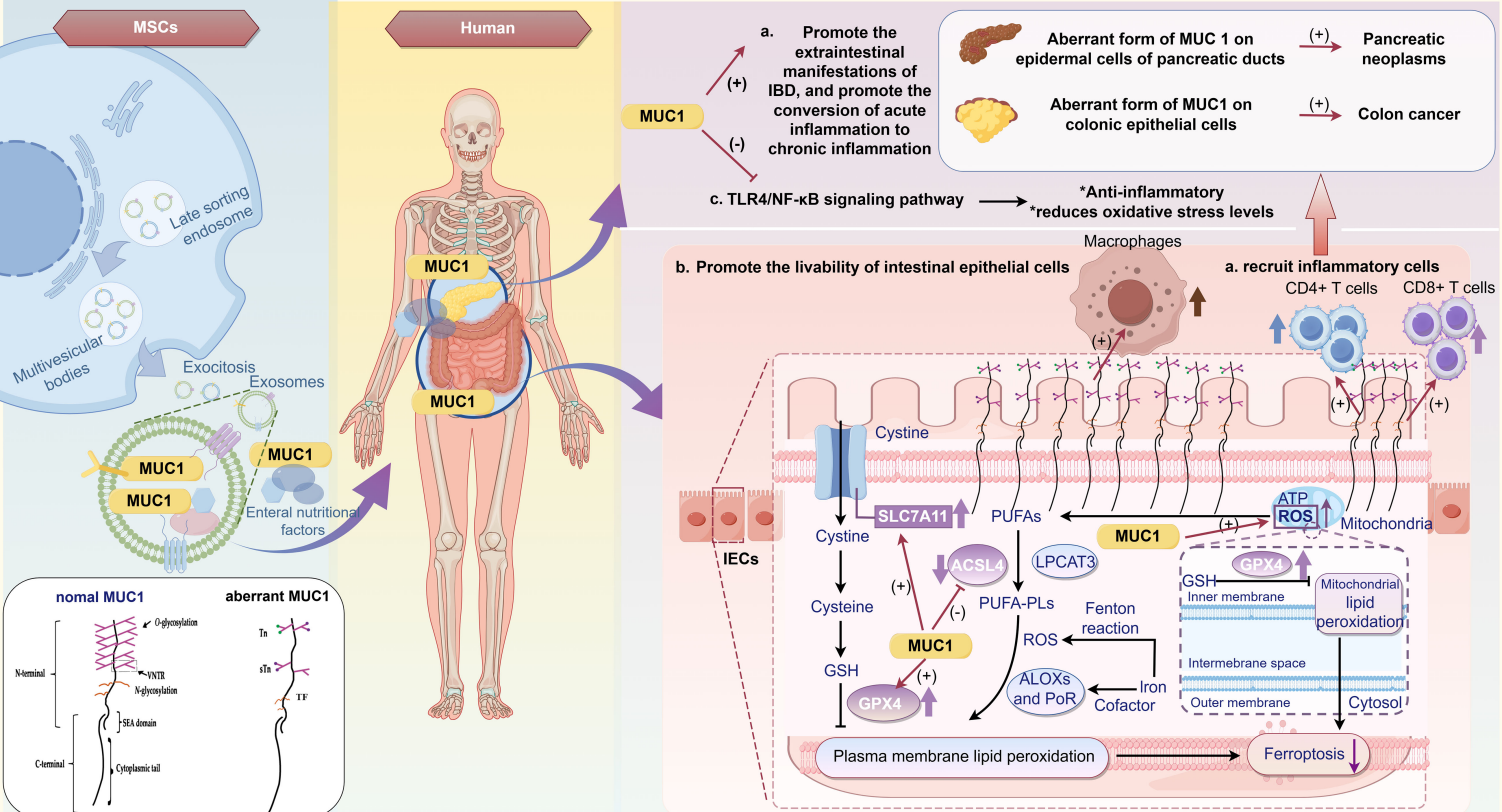
Role of the apoptosis-related gene/tumor-associated antigen MUC1 in mesenchymal stem cell-mediated treatment of IBD. MSCs significantly upregulate the expression of MUC1, which is closely associated with the pathological processes of IBD, by preventing disruption of antioxidant defenses, regulating the sensitivity of cell ferroptosis, and improving inflammation and extraintestinal manifestations of IBD: **(A)** MUC1 is a tumor-associated antigen that is downregulated in IBD. The presence of abnormal forms of MUC1 has a profound impact on the timing and severity of inflammation, as well as the progression of certain extraintestinal manifestations of IBD. The low-glycosylated and overexpressed MUC1 can attract innate immune system cells, including CD4^+^ and CD8^+^ T cells, immature myeloid DCs, and macrophages, thereby promoting acute inflammation. Over time, acute inflammation can evolve into chronic inflammation. The presence of abnormal forms of MUC1 can also enhance the carcinogenic potential of colon and pancreatic cells. **(B)** MSCs can secrete various intestinal trophic factors and exosomes to inhibit intestinal epithelial cell death. Among these, MUC1 may promote regeneration of the epithelial cell barrier by inhibiting ferroptosis. MUC1 can upregulate ferroptosis-related genes, such as the glutamate/cystine antiporter SLC7A11 and GPX4 in small intestinal crypt epithelial cells while downregulating ACSL4 to facilitate epithelial cell growth. **(C)** MUC1 has an anti-inflammatory effect by targeting the TLR4/NF-κB signaling pathway and reducing oxidative stress levels. IBD, inflammatory bowel disease; MSCs, Mesenchymal Stromal/Stem Cells; DCs, dendritic cells; IEC, intestinal epithelial cells; MUC1, Mucin 1; CD, Cluster of Differentiation; SLC 7A11, Solute carrier family 7 member 11; GPX 4, Glutathione peroxidase 4; ACSL 4, Acyl-CoA Synthetase Long Chain Family Member 4;TLR, Toll-like receptor; NF-κb, Nuclear factor- kappa B; GSH, Glutathione; PUFAS, Polyunsaturated Fatty Acids; ATP, Adenosine Triphosphate; ROS, Reactive Oxygen Species; LPCAT, Lysophosphatidylcholine Acyltransferase 3; PLs, Phospholipids; ALOXs, Arachidonate Lipoxygenases; PoR, P450 Oxidoreductase.

The sensitivity of MSCs to prevent the destruction of antioxidant defenses and ferroptosis in inflammatory colitis is closely related to the pathological process of IBD. MSCs inhibit IEC death by secreting various intestinal trophic factors and exosomes. Transcriptome analysis showed that MUC1, an apoptosis-related gene, is involved in IBD treatment using MSCs. Additionally, MUC1 may serve as a marker of iron toxicity in UC and may regulate cellular iron toxicity (288). A comprehensive analysis of iron-related genes revealed that MUC1 inhibition results in ROS accumulation and redox homeostasis disruption. Furthermore, MUC1 was found to be essential for the growth and regeneration of IECs when the MUC1 inhibitor GO-203 was used. Mechanistically, MUC1 exerts anti-inflammatory effects and reduces oxidative stress levels by targeting the TLR4/NF-κB signaling pathway (289). UC-MSCs protect intestinal barrier function by significantly modulating MUC1 and iron deposition regulators. These results indicate that MSCs can facilitate recovery from IBD and enhance the functionality of the intestinal mucosal barrier by upregulating MUC1, inhibiting cell death and preventing ferroptosis. MUC1 upregulation is crucial in IBD treatment using MSCs and apoptosis (236). Conversely, Muc1 KO mice have been reported to be resistant to DSS-induced colitis, possibly due to enhanced mucus barrier function or reduced T cell recruitment to the affected site (290). The apoptosis-related gene MUC1 has been implicated in the MSC-mediated treatment of IBD (**Figure 6**). However, the regulatory role of MSCs in abnormal MUC1 forms remains experimentally unverified, highlighting the need for further research. MUC1 is reported to be downregulated in patients with IBD (78), and its expression levels are low in the peripheral blood of MDSCs in both humans and mice (235). This may explain the previously described relationship between MDSCs and IBD. Furthermore, as previously discussed, MUC1 also exerts a regulatory effect on MDSCs. Overall, these findings suggest that MUC1 may serve as a key regulatory molecule for MSCs within the IBD microenvironment.

## 10 Challenges and future perspectives

The mechanisms by which MSCs treat IBD are diverse, and therapeutic approaches vary; however, several challenges remain unaddressed. First, MSCs demonstrate distinct characteristics in preclinical studies, which are influenced by variations in tissue sources, donor factors, and culture conditions. As previously discussed, MSCs are substantially altered by inflammatory or anti-inflammatory stimuli, making it difficult to precisely determine how MSC variability affects their capacity to induce immunomodulatory effects. Despite the extensive and nuanced regulatory roles of MSCs in the immune microenvironment of IBD, no study has fully elucidated their comprehensive regulatory effects. Furthermore, the diverse secretomes of living MSCs may contribute to more complex immune regulatory mechanisms. For example, in a rhesus monkey model, intracranial transplantation of allogeneic BM-MSCs was found to be less immunogenic than that of autologous BM-MSCs. Consequently, long-term transplantation may lead to the host developing an immune response against MSCs, negatively affecting the durability of the engraftment (291). Second, considerable differences in phenotype and disease severity among patients with IBD pose a significant challenge in predicting MSC treatment outcomes and responses (292). In a clinical trial, patients with CD showed a marked improvement following UC-MSC infusion. However, most patients still required adjuvant therapy with other agents at 12 months, suggesting that MSC therapy may not be superior to TNF blockers in long-term efficacy (293).

Nevertheless, MSC therapy remains a viable option for many patients with IBD who do not respond adequately to other treatments, owing to the unique and broad immunomodulatory effects of MSCs.

Acellular therapy for patients utilizing MSC-Exos overcomes the limitations of cell replacement therapy, including immunogenicity, cargo specificity, and broad biological regulatory effects. However, their applications are limited by several challenges. The MSC source significantly affects the composition and biological functions of MSC-Exos, resulting in variability in their immunoregulatory capabilities, ease of isolation, and therapeutic efficacy (294). For instance, adipose-derived MSC-Exos are frequently used in IBD research because of their favorable immunomodulatory characteristics and relative ease of isolation. However, their ability to efficiently target inflamed tissues is inferior to that of exosomes derived from human BM-MSCs. Additionally, MSCs obtained from the tonsils of young donors (tonsil-derived MSCs) demonstrate superior proliferation rates compared to MSCs isolated from other tissue sources. Their enhanced capacity to differentiate into intestinal stem cell-like cells positions tonsil-derived MSCs as promising for IBD (295). Given the inherent complexity of IBD pathogenesis, careful selection of MSC-Exos tailored to individual patient immune dysregulation profiles and specific intestinal conditions is essential to optimize therapeutic outcomes.

Moreover, the route of exosome administration significantly affects their bioavailability and therapeutic efficacy. In the harsh gastrointestinal environment, characterized by digestive enzymes, pH fluctuations, and dense biological barriers, oral administration may lead to structural degradation of exosomes and premature release of their payload, thereby reducing their bioavailability (296). Intravenous injection enables the direct delivery of exosomes into the systemic circulation, circumventing gastrointestinal degradation; however, this method has limitations. Exosomes may be rapidly cleared by the mononuclear phagocytic system, leading to reduced accumulation in target organs (297). One of the therapeutic goals of IBD treatment is the localized delivery of therapeutic agents to the inflamed intestinal mucosa to minimize systemic side effects. Numerous ongoing studies are exploring Nanoparticles-based encapsulation strategies for targeted exosome delivery, which offer promising prospects for achieving this objective (298).

Although the homing ability of MSC-Exos allows them to specifically migrate to inflamed regions, such as intestinal inflammation in IBD, and exert therapeutic effects, enhancing their enrichment at these sites remains a challenge. Various strategies, including genetic engineering, surface modification, and tissue engineering, can be employed to modify MSCs such that the exosomes they produce carry ligands capable of specifically binding to inflamed tissues. These ligands may include antibody fragments or specific peptides, which improve innate homing ability and enable targeted delivery to specific tissues or organs, thereby enhancing exosome accumulation at inflammation sites (299). Additionally, cell membrane coating techniques can be applied to modify exosomes (300), or MSC-Exos can be combined with biological scaffolds to create EV-based microcarriers with bioadhesive properties (301), improving their accumulation at intestinal inflammation sites and effectively controlling exosome release, thereby increasing their retention time in the gut.

Another challenge is the discrepancy between *in vitro* and *in vivo* findings in MSC studies and the negative impact of allogeneic or xenotransplantation on therapeutic efficacy. For example, osteoblasts differentiated *in vitro* from MSCs in the New Zealand white rabbit osteogenic model retained their immune privileges and immunomodulatory properties *in vitro* but lost their immunomodulatory properties after transplantation (302). These differences suggest that careful monitoring of MSC immunogenicity is essential when transitioning from *in vitro* to *in vivo* models and for ensuring durable therapeutic effects in clinical applications.

MSC-Exos are generally regarded as a low-immunogenic therapeutic strategy, offering enhanced safety and promising therapeutic outcomes in the treatment of IBD compared to traditional drugs and MSC-based therapies (303). However, MSC-Exos may carry donor-specific antigens such as HLA, which could trigger immune responses in recipients (304). Additionally, viruses can exploit the exosome biogenesis pathway by using exosomes as vectors for transmission (305). Therefore, comprehensive safety evaluations, including immunological and pathogen screening, are essential prior to the clinical use of MSC-Exos for therapeutic applications (306). Moreover, long-term toxicity studies are necessary to confirm their safety profiles (307). Strict control of the preparation process, removal of exogenous substances, and reduction of immunogenicity are essential strategies for minimizing the immunogenic potential of MSC-Exos. Additionally, genetic engineering or chemical modifications can be employed to lower the immunogenicity of MSC-Exos while enhancing their immunoregulatory functions (299). Currently, a combination of multiple isolation methods is recommended, such as initial ultracentrifugation followed by further purification using size-exclusion chromatography or affinity chromatography. Culturing conditions may also be optimized, such as providing inflammatory stimuli (308), and optimal storage methods are recommended, including storage at -80°C in buffers containing protective agents such as trehalose or glycerol (309). These measures could enhance the immunosuppressive properties and biological activity of MSC-Exos, thereby improving their therapeutic efficacy against IBD.

We found that TR-MSC function is impaired in patients with IBD, which may limit their ability to perform tissue repair and immune regulation. Experimental findings indicate that the stem cell function of CD MSCs is lost owing to abnormal differentiation in UC. This provides a strong rationale for the use of allogeneic MSCs derived from healthy donors as therapy for active CD (134). Further research is required to elucidate the mechanisms underlying TR-MSC injury and its role in IBD.

Although MSCs are highly effective at regulating the immune microenvironment, future studies should investigate the impact of additional factors or chronic inflammation on MSC-mediated immunomodulation and tissue regeneration. However, the role of TR-MSCs IBD progression remains poorly understood. Furthermore, future work should focus on optimizing MSC and MSC-Exos treatment strategies, encompassing aspects such as cell dose, route of administration, treatment frequency, and patient selection criteria, to minimize variations in MSC paracrine and therapeutic efficacy, particularly in terms of clinical translatability. Developing therapeutic strategies that target specific immune cells or molecules is needed to improve the specificity and efficacy of MSC therapy, including the use of CRISPR/Cas9 gene-editing technology to enhance the immunomodulatory functions of MSCs or improve their targeting in IBD treatment. MSCs infected with lentiviruses encoding CX3C chemokine receptor 1 (CX3CR1) and IL-25 can be better manipulated to deliver them to the inflamed colon and enhance their immunosuppressive capacity (261). More clinical trials and real-world data are necessary to evaluate the efficacy and safety of MSCs in IBD treatment. Ultimately, exploring combinations of MSCs with other therapeutic approaches, such as biologics, nutritional therapy, or surgery; enhancing immunomodulatory potential, homing mechanisms, and improved cryopreservation techniques; and conducting rigorous preclinical and clinical studies could achieve a more comprehensive therapeutic effect. We believe that MSCs and MSC-Exos have great potential for future applications in the clinical setting of IBD.

## 11 Conclusion

We found that the interaction between immune cells and MSCs mitigates the occurrence and progression of IBD and is beneficial for improving its complications. MSCs and MSC-Exos can modulate the immune microenvironment in IBD by targeting various immune cells, including macrophages, NK cells, DCs, T lymphocytes, B lymphocytes, neutrophils, and MDSC. The main mechanisms of MSC immunomodulation involve cell–cell contact and paracrine activities initiated by EVs, cytokines, chemokines, inflammatory stimuli, or co-culture with other cells. Additionally, MSCs contribute to intestinal flora enhancement, intestinal barrier repair, and fibrosis reduction owing to their significant impact on the immune microenvironment. We also proposed key immune targets for MSCs in the context of IBD at the tissue, cellular, and molecular levels, as recent discoveries indicate that MSCs can be modified through genetic engineering.Compared to traditional drugs and biological therapies, MSC-based treatments hold promise as a significant biological system, offering enhanced therapeutic effects and improving the quality of life of patients with IBD in clinical settings.

## Glossary

AT-MSCs: Adipose Tissue-Derived Mesenchymal Stromal/Stem Cells
BM-MSCs: Bone Marrow-Derived Mesenchymal Stromal/Stem Cells
CAC: Colitis-Associated Colon Cancer
CD: Crohn’s Disease
DCs: Dendritic Cells
DSS: Dextran Sulfate Sodium
ECM: Extracellular Matrix
EVs: Extracellular Vesicles
GF: Germ-Free
HA: Hemagglutinin
HIF-1α: Hypoxia-Inducible Factor 1-Alpha
hBM-MSCs: Human Bone Marrow-Derived Mesenchymal Stromal/Stem Cells
hMSCs: Human Mesenchymal Stromal/Stem Cells
hUC-MSCs: Human Umbilical Cord-Derived Mesenchymal Stromal/Stem Cells
IECs: Intestinal Epithelial Cells
IL: Interleukin
IFN-γ: Interferon-gamma
Lgr5: Leucine-Rich Repeat–Containing G-Protein Coupled Receptor 5
MDSCs: Myeloid-Derived Suppressor Cells
MUC1: Mucin 1
Muc1-KO: Mucin 1 Knockout
MSCs: Mesenchymal Stromal/Stem Cells
MSC-Exos: MSC–Derived Exosomes
NLRP3: NOD-Like Receptor Protein 3
NK: Natural Killer Cell
ROS: Reactive Oxygen Species
TNF-α: Tumor Necrosis Factor-Alpha
TNBS: 2,4,6-Trinitrobenzenesulfonic Acid
TR-MSC(s): Tissue-Resident Mesenchymal Stromal/Stem Cell(s)
UC: Ulcerative Colitis
UC-MSCs: Umbilical Cord-Derived Mesenchymal Stromal/Stem Cells
VNTR: Variable Number Tandem Repeat
